# Cancer driver mutation prediction through Bayesian integration of multi-omic data

**DOI:** 10.1371/journal.pone.0196939

**Published:** 2018-05-08

**Authors:** Zixing Wang, Kwok-Shing Ng, Tenghui Chen, Tae-Beom Kim, Fang Wang, Kenna Shaw, Kenneth L. Scott, Funda Meric-Bernstam, Gordon B. Mills, Ken Chen

**Affiliations:** 1 Department of Bioinformatics and Computational Biology, The University of Texas M.D. Anderson Cancer Center, Houston, Texas, United States of America; 2 Institute for Personalized Cancer Therapy, The University of Texas M.D. Anderson Cancer Center, Houston, Texas, United States of America; 3 Department of Human and Molecular Genetics, Baylor College of Medicine, Houston, Texas, United States of America; 4 Department of Investigational Cancer Therapy, The University of Texas M.D. Anderson Cancer Center, Houston, Texas, United States of America; 5 Department of Systems Biology, The University of Texas M.D. Anderson Cancer Center, Houston, Texas, United States of America; Johns Hopkins University, UNITED STATES

## Abstract

Identification of cancer driver mutations is critical for advancing cancer research and personalized medicine. Due to inter-tumor genetic heterogeneity, many driver mutations occur at low frequencies, which make it challenging to distinguish them from passenger mutations. Here, we show that a novel Bayesian hierarchical modeling approach, named rDriver can achieve enhanced prediction accuracy by identifying mutations that not only have high functional impact scores but also are associated with systemic variation in gene expression levels. In examining 3,080 tumor samples from 8 cancer types in The Cancer Genome Atlas, rDriver predicted 1,389 driver mutations. Compared with existing tools, rDriver identified more low frequency mutations associated with lineage specific functional properties, timing of occurrence and patient survival. Evaluation of rDriver predictions using engineered cell-line models resulted in a positive predictive value of 0.94 in *PIK3CA* genes. Our study highlights the importance of integrating multi-omic data in predicting cancer driver mutations and provides a statistically rigorous solution for cancer target discovery and development.

## Introduction

Large-scale cancer genome projects, such as The Cancer Genome Altas (TCGA), and the International Cancer genome Consortium (ICGC) have systematically catalogued hundreds of thousands of somatic mutations in a wide variety of adult and pediatric cancers. However, the functional significance of the majority of these mutations remains unknown. As implicated in previous studies, only a small fraction of genetic alterations are expected to be driver mutations that functionally drive the malignancy of tumor cells and the rest are likely passenger mutations conferring no selective advantage [1]. Distinguishing driver mutations from passenger mutations remains one of the most pressing challenges in ongoing cancer genomic research [2].

Many computational approaches have been developed over years for predicting cancer drivers at gene and/or mutation levels [3–9]. Many of them rely on examining mutational frequencies, which are indicative of the fitness of a mutant allele and its oncogenic function. Unfortunately, the majority of mutations in tumors occur rather infrequently, making frequency-based approaches marginally effective. In a separate vein, approaches such as SIFT [10] and PolyPhen [11] compute a phenomenological score for each mutation based on the physical-chemical properties or the extent of evolutionary conservation of the amino acid sequences affected by the mutation. OncodriveFM and FunSeq search for positive selection and purifying selection patterns to identify cancer driver genes [4, 12]. These approaches focus on local sequence features, missing the gene regulatory effect associated with individual mutations. Modeling somatic mutations in the context of genome-wide mRNA and/or protein expression levels can quantitatively improve the accuracy of driver mutation prediction [13]. It has been shown that integrating quantitative trait data such as mRNA expression levels can lead to more accurate identification of disease-causing polymorphisms in genetic disorders [14, 15]. However, benefits of integrating expression data in predicting cancer driver mutations have not been fully explored, lacking are approaches that systematically associate driver mutations with genome-wide expression patterns. Statistical challenges may exist in modeling excessive heterogeneity in somatic mutation data.

Some recent studies have started to explore transcriptome data for systematic prediction of cancer drivers. These studies are limited in several important ways. Methods such as MOCA [16] and DriverNet [17] examined mutations at gene levels, ignoring the functional difference of the mutations within a gene. Methods such as xSeq [18] measured the effects of individual mutations in gene expression through an *in silico* model of known pathway networks. However, global and novel regulatory effects unaccounted for by the predefined networks could be missed, leading to biased inference. Indeed, it has been demonstrated that many cancer mutations are neomorphic with unexpected functionality [19]. Importantly, these methods do not integrate known evolutionary and structural properties of mutations characterized by functional impact scores (FISs) generated by programs such as GERP [20] and SIFT [21], which have been shown to be very informative at identifying functional variants. Increasingly more projects such as TCGA and ICGC now generate multi-omic data from the same tumor samples. A growing need exists to integrate multi-omic data towards more accurate identification of driver mutations.

Here, we hypothesize that a subset of driver mutations not only have high FISs but also significantly affect mRNA/protein expression levels in tumor samples, as systemic alternation in mRNA/protein expression levels are often required for acquisition of new distinct phenotypes, such as inhibition of apoptosis, increased cell proliferation, acquired resistance to therapy, and adaptation to local microenvironments [22]. If our hypothesis is correct, a computational method that systematically integrates FIS, and mRNA/protein expressions shall lead to enhanced discovery of that subset of driver mutations, particularly those occurring at low-frequency but associated with systemic changes in expression levels.

## Results

### rDriver: An integrative approach to predict driver mutation

**Fig 1:**
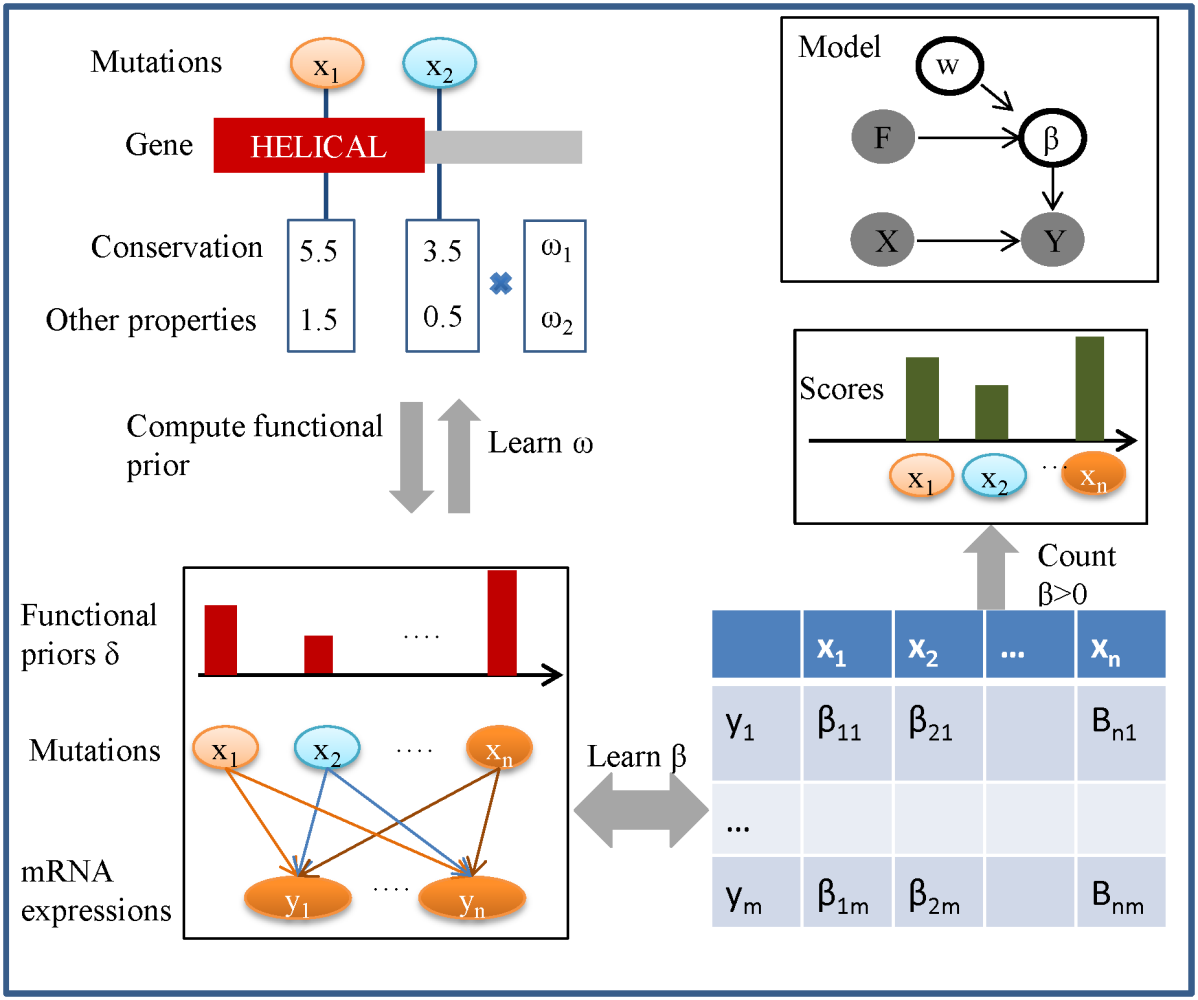
Outline of rDriver. We define a Bayesian hierarchical model that predict mRNA expression levels from mutations and related functional genomic annotations. The regulatory features, such as the evolutionary conservation or the physiochemical properties of a mutation are integrated into the model by a weight prior vector w. The program proceeds to learn these parameters by iterating the following three steps: (i) rDriver takes as input the regulatory priors for each mutation, and constructs a set of regularized penalty δ for the mutation. In the first iteration, the regulatory priors are assumed to be uniform. (ii) rDriver takes as input the mutations X and their specific regularized penalty to learns the regression coefficients β, representing the predictability of a mRNA expression level from a mutation. (iii) rDriver takes as input the output of the previous steps and updates the regulatory prior parameter of each mutation through minimization of the objective function. The final converged solution will result in a mutation and expression association matrix. The likelihood (score) that a mutation is a driver is computed based on the number of non-zero regression coefficients between the mutation and the set of mRNAs, followed by permutation tests.

We developed a rDriver approach, which predicts driver mutations based on genome-wide mRNA/protein expression levels, and the FISs of individual mutations (Fig 1 and Methods). The statistical framework of rDriver was motivated by a Bayesian framework developed for performing eQTL mapping [23], which identifies the x variables (mutations) that best predict the *y* variables (expressions). The regression coefficients β are regularized by prior score δ, which quantifies a prior functional potential of each mutation as a weighted summation of a set of FISs, such as GERP and SIFT scores. Under this model, all the mutations are assessed simultaneously to account for potential synergistic or antagonistic effects among mutations. Due to the sparse nature of the associations (i.e., regulatory mutations are rare), most regression coefficients shall shrink to zero under iterative L1 regularization. Only those that represent robust associations retain non-zero values. This process does continuous shrinkage of associations and automatic variable selection simultaneously. It results in an association matrix that pairs each mutation with each gene. A driver likelihood score is computed for each mutation based on the number of genes associated with the mutation in the association matrix. The larger the score is, the more likely the mutation is a driver. A p-value is further computed for each mutation based on empirical distribution of the driver scores (Methods), reflecting the probability that the observed amount of association was not produced by chance.

### Assessment of rDriver using the TCGA breast carcinoma (BRCA) data

**Fig 2:**
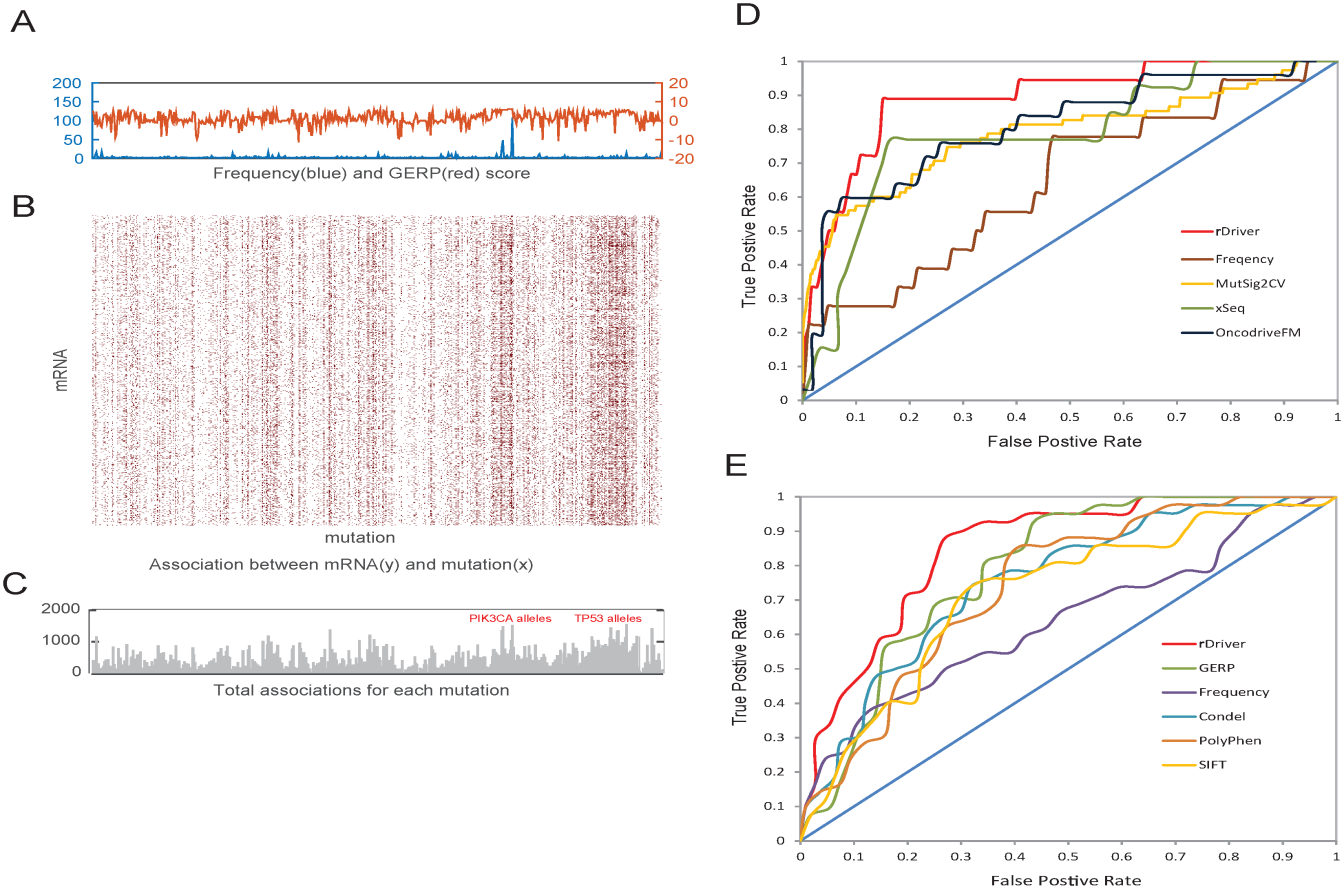
rDriver output of BRCA and its comparison with other methods. (A) the frequency (blue) and GERP score (red) distribution across the 528 mutations in TCGA BRCA data (the SIFT score is not shown due to missing value) (B) the association matrix between the mRNAs and the mutations, with brown dots representing non-zero association coefficients. (C) the total number of non-zero values in the association matrix column-wise, representing the likelihood of driver mutations. A few known driver mutation hotspots in *PIK3CA* and *TP53* are labelled (red text). (D) Receiver operator characteristic (ROC) curves comparing the sets of driver genes predicted by various programs against a set of 17 known cancer driver genes in the Cancer Gene Census. (E) ROC curves comparing the sets of mutations predicted by rDriver, frequency, GERP, Condel, SIFT and PolyPhen against a set of 42 known driver mutations.

We examined rDriver using TCGA BRCA data that included 752 breast carcinoma patients. We focused on 544 mutations in these patients that occurred at least twice in the cohort (Fig 2A). To reduce dimensionality, we limited mRNA expression data to a subset of 3,030 genes (S1 Dataset), which included transcription factors, chromatin remodelers, and signal transduction genes, known to play important roles in cancer [24]. The GERP and SIFT scores are used to calculate the prior functional potential for each mutation. The SIFT scores were available for only single-nucleotide variants (SNV) and the GERPs for both SNVs and small insertion-deletions (indel) existing in the mutation data. We assigned the missed SIFT scores of indel mutations with the average value of the SNVs available.

In total, rDriver predicted 228 putative driver mutations and 316 putative passenger mutations in the BRCA data (S2 Dataset) and estimated an association matrix between the mutations and mRNA expression levels (Fig 2B). The resulting driver scores demonstrated substantial variation across different mutations (Fig 2C). An average of 26% mRNA expression variations were explained by the mutations (S1 Fig). Mutant alleles in known driver genes such as *TP53* and *PIK3CA* had considerably higher scores than others. We found that the rDriver predictions were largely consistent with results obtained independently from differential gene expression analysis (Methods), where the genes which are differentially expressed between samples with and without a specific mutation are identified. For example, top-scoring mutations in *PIK3CA*, *GATA3*, *AKT1*, and *TP53* were associated with a large number of differentially expressed genes (DEGs), whereas bottom-scoring mutations in *AOAH*, *FAM157B* and *NCOA3* were associated with few or no DEGs (Panel A in S2 Fig). The rank correlation between rDriver score and the number of DEG associated are 0.82 (p = 0.002) based on the top 9 most frequent mutations in BRCA (Panel B in S2 Fig). Pathway analysis of the associated genes for each mutation revealed enrichment of a range of functions (S3 Dataset). The top-scoring mutations turn to associate with genes in cell cycle and cancer related pathways (S3 Fig). These results indicated that our algorithm has captured the transcriptional regulatory effects of somatic mutations in mRNA expression levels and can potentially enhance the discovery of driver mutations involved in transcriptional regulation.

We further assessed the accuracy of rDriver predictions by comparing them with cancer drivers known in the literature and with those predicted by other algorithms. First, we compared the sets of predicted driver genes against a set of 17 highly probable cancer genes that were listed in the cancer gene census (CGC) [25, 26] and were mutated in our set (S4 Dataset). A gene is predicted as a driver gene if it contains one or more predicted driver mutations. In this benchmark, rDriver achieved the best classification accuracy with an area under curve (AUC) of 0.89 (Fig 2D), followed by OncodriveFM (AUC = 0.79), xSeq (AUC = 0.78) and MutSig2CV [9] (AUC = 0.77). An approach based purely on mutation frequency (Methods) performed poorly (AUC = 0.63). Similar trends were observed (S4 Fig) when we compared predictions against another list of 18 cancer genes (S4 Dataset) [27] mutated in our data. Second, we compared the sets of predicted driver mutations with a list of 42 missense SNVs that were known cancer drivers [28]. Only missense SNVs were compared due to restrictions of some of the predictors. rDriver was able to achieve evidently better classification performance (AUC = 0.85) on this set than other predictors (Fig 2E), followed by GERP (AUC = 0.78), Condel (AUC = 0.75), SIFT, PolyPhen and a frequency based approach (Methods).

### Identifying cancer context specific candidate driver mutation across the TCGA data

Encouraged by our results on the BRCA data, we further expanded rDriver analysis to a TCGA Pan-cancer cohort (S1 Table), which included 9,938 recurrent mutations from a total 2,284 patients across 8 cancer types. In total, rDriver predicted 1,389 putative driver mutations (S5 Dataset) in 951 genes across the cancer types, which explained an average of 28% variance in mRNA expression levels. Seven of the eight cancer types each had over 100 putative driver mutations except for the GBM. Most of patient carried at least 1 putative driver mutation.

**Fig 3:**
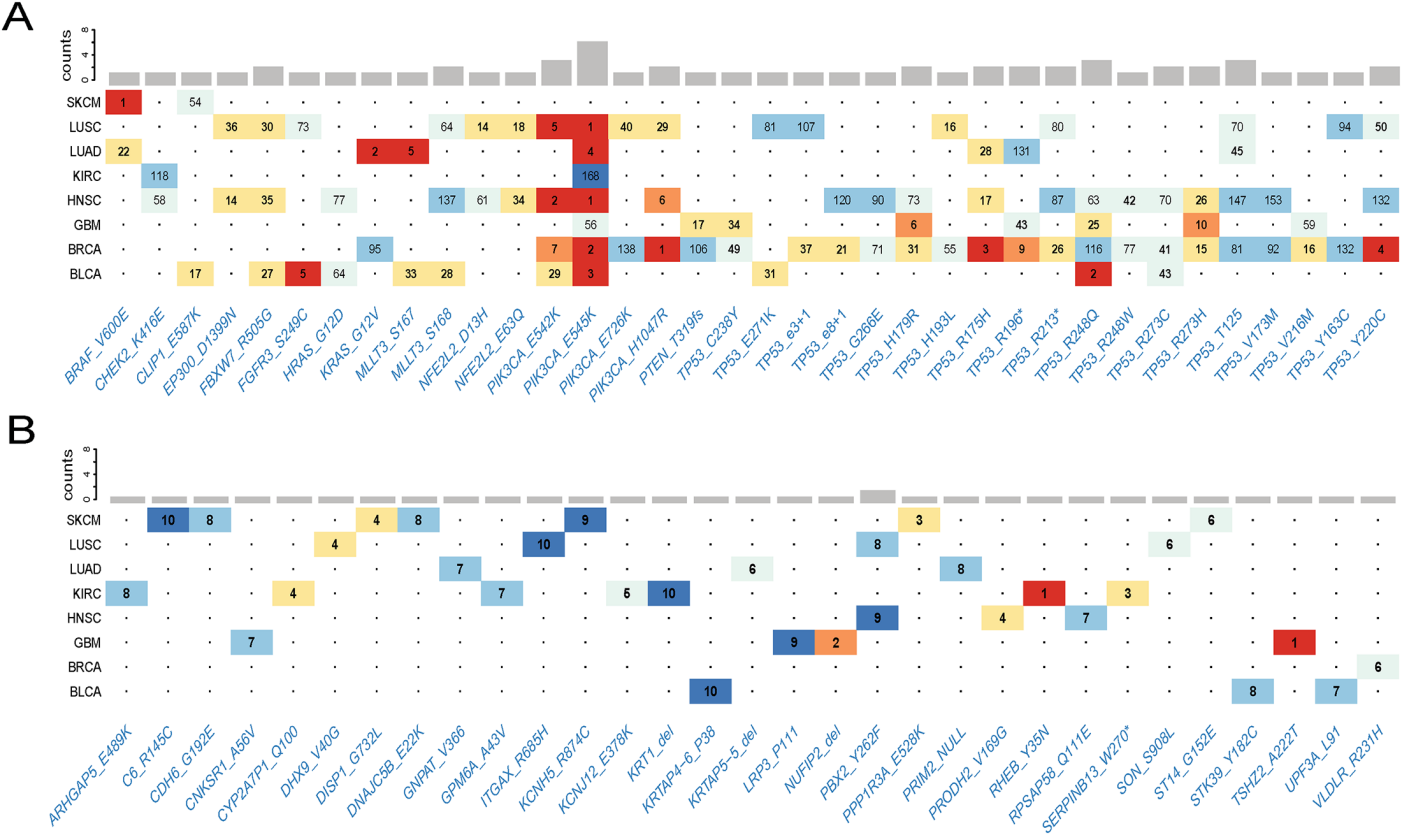
Functional annotations of rDriver predictions in 8 cancer types. (A) The predictedat drivers present in cgc and at least 2 cancer types. (B)The predicted top 10 ranked drivers in novel genes. The color represents different rank range.

Most affected genes possessed only one putative driver mutation. A subset (143) of genes such as *PIK3CA*, *TP53*, *VHL* and *EGFR*, possessed two or more driver mutations (Panel A in S5 Fig). Most of the putative driver mutations appeared in a single tumor type (Panel B in S5 Fig). Only a relatively small number occurred in multiple cancer types. For example, *PIK3CA* E545K was predicted in 7 cancer types. 302 of the 1,389 predicted driver mutations in 95 of the CGC genes demonstrated striking cancer-type specificity, and 16 of them are predicted at least 2 cancer types (Fig 3A). Some of mutation alleles were ranked highly in one cancer type, but not in the others. For example, *BRAF* V600E ranked the 1st in SKCM, 22th in LUAD. *PIK3CA* E545K ranked among top 5 in 5 cancer types, but very low in KIRC and GBM. These results indicated context specific nature of rDriver predictions due largely to dynamic mRNA expression profiles in different cancer types.

For simple illustration of novel discovery, we extracted the top 10 rank of each cancer type and obtained 32 mutant alleles resided in 31 non-CGC genes (Fig 3B). In searching the literature, we found strong connections of at least 15 of these genes to known cancer genes **(**S2 Table**)**. The roles of several of these genes (for example, *CDH6* and *TSHZ2*) have recently been elucidated. Three of them (*RHEB*, *PPP1R3A* and *CNKSR1*) encode proteins in insulin signaling pathway involved in glucose and lipid homeostasis. Two (*DHX9* and *UPF3a*) have a role in the maintenance of genomic and transcriptomic stability. Three (*ITGAX*, *STK39* and *C6*) are found to be associated with aggressive cancer in GWAS and QTL studies. Further investigation revealed that 26/31 of the genes were known interaction partners of previous characterized cancer genes, based on the Pathway Common database [29].

We further compared driver mutations predicted by rDriver and mutation frequencies with driver mutations known in the literature and occurred at least twice in each of the tumor types. We found that rDriver were the most accurate algorithm in 8 cancer types (S6 Fig).

### Timing of the rDriver predicted driver mutations

**Fig 4:**
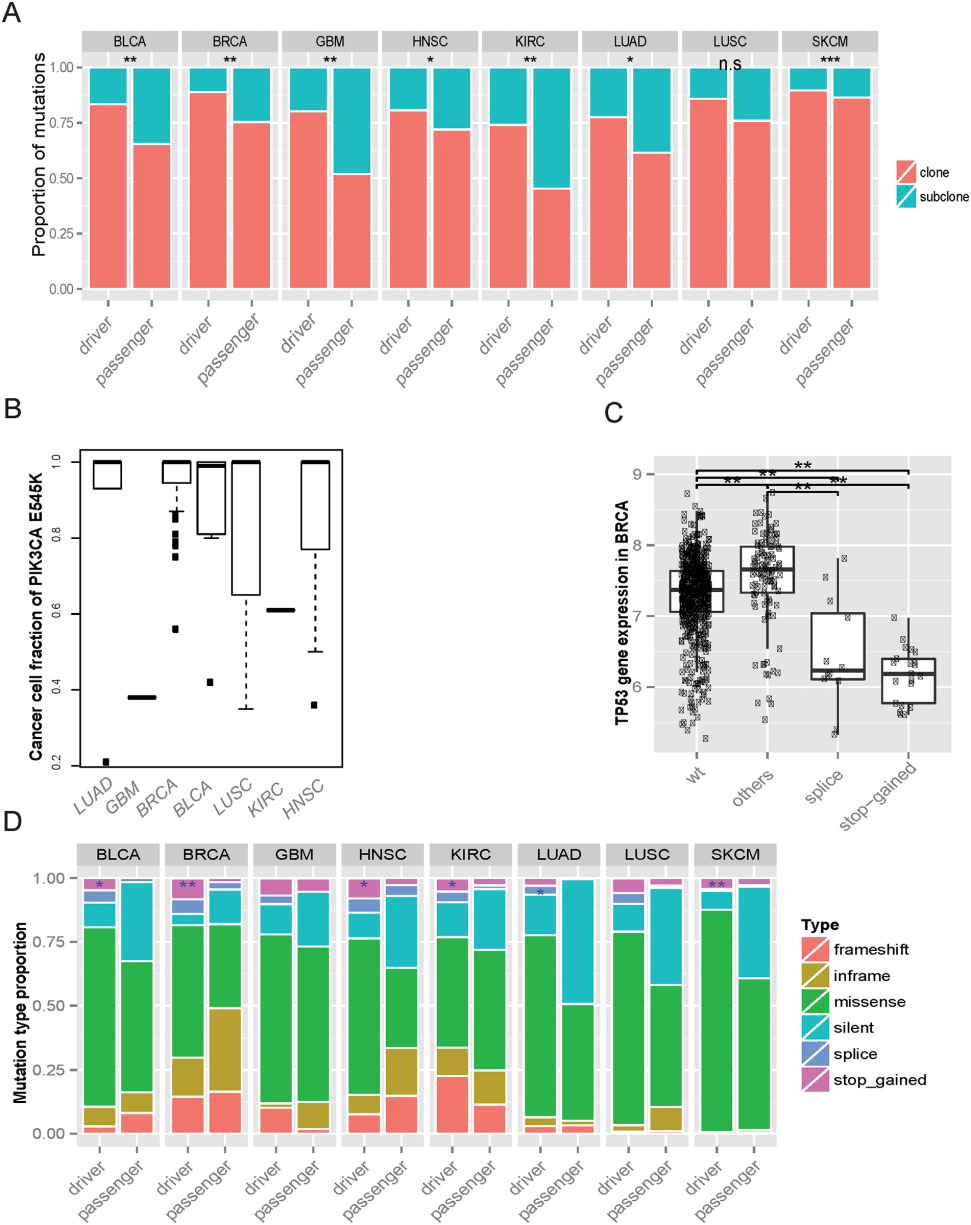
Clone and subtype analysis. (A) The proportion of clonal/subclonal in driver and passenger group for each cancer types. Significance from Fisher’s exact test is indicated. Exact p values are as follows; BLCA, p = 4.53e-03; HNSC, p = 4.70e-02; SKCM, p = 1.46e-05; GBM, p = 5.45e-03; BRCA, p = 2.89e-03, LUSC, p = 6.686e-02; KIRC, p = 2.62e-03, LUAD, p = 1.372e-02 by Chi-squared test. (B) The fraction of cancer cells mutated for *PIK3CA* E545K and *BRAF* V600E in effected cancers. (C) The distribution of mutation subtypes in the driver and the passenger groups predicted by rDriver. Stars indicate significance of enrichment of stop-gained or splice related variants in driver group. (**<0.01, *p<0.05; Fisher’s exact test) (D) Altered *TP53* gene expression associated with different types of mutations in BRCA. (**<0.01, *p<0.05; t-test).

As it is known the driver mutations tend to be initiators of tumorigenesis, likely acquired earlier than other mutations and were therefore more clonal (i.e., present in more cancer cells). We next did post-processing analysis of timing of the rDriver predicted mutation. We obtained estimated cancer cell fractions (CCF) and clonal/sub-clonal classification for most of the mutations from a previous study [30]. Overall, there are significantly more clonal mutations in the rDriver predicted driver groups than in the passenger groups (Fig 4A, Methods) in almost all the cancer types, except for LUSC that had fewer mutations with estimated CCFs (S3 Table) (all p < 0.05 except LUSC, Fisher’s exact test). The driver mutations had significantly higher CCFs than did the passenger mutations (S7 Fig). Particularly, we found that 93% of the top 10 predicted drivers in the 8 cancer types were clonal mutations. Among them were novel mutations in *VLDLR*, *CNKSR1*, *RHEB*, *CDH6*, *ST14*, *PPP1R3A*, and *ITGAX*. These independently derived CCF data supported the overall validity of our results.

We also found that the clonality of the mutations varied considerably across different cancer types. For instance, *PIK3CA* E545K was predominantly clonal with high cancer cell fractions in LUAD, BRCA, BLCA, LUSC and HNSC, but was often sub-clonal in GBM and KIRC (Fig 4B). Such varying CCFs in different cancer types might reflect the context specific functionality of these driver mutations. For example, a GBM patient had a *PIK3CA* E545K mutation and an *EGFR* A224V mutation, both of which were predicted as driver mutations. However, the *EGFR* mutation was estimated to be present in all the cancer cells, whereas the *PIK3CA* mutation was estimated to be present in only 38% of the cancer cells (S8 Fig). As a receptor tyrosine kinase (RTKs) in the upstream of the PI3K pathway, *EGFR* is able to activate the PI3K pathway. The difference in the CCFs of these two driver mutations postulated that the *EGFR* mutation might drive tumor initiation, whereas the *PIK3CA* mutation contributed to tumor progression. Similar pattern was observed in a KIRC patient with co-occurring *PIK3CA* E545K and *VHL* mutation (S8 Fig). Interestingly, rDriver assigned lower ranks to *PIK3CA* E545K in GBM and in KIRC than in other cancer types, consistently with the observed CCF patterns.

### rDriver identifies subtype specific mutations

We examined the mutation types in the rDriver predicted driver group, as contrast to those in the passenger group. The driver group appeared to contain a greater proportion of stop-gained and splice-site mutations, but less in-frame or silent mutations than the passenger group in all of the 8 cancer types (Fig 4D). Most affected genes have only one stop-gained or splice-site mutation. However, tumor suppressor genes such as *TP53* had 10 alleles in BRCA (S2 Dataset). Different mutation subtypes in a gene may indicate their context-varying functions. A stop-gained mutation may lead to protein truncation, mRNA degradation via Nonsense-Mediated Decay (NMD) [31] or dominant negative phenotype; while a splice-site mutation may not only lead to NMD, but also create protein isoforms of differing, even opposing functions [32]. Interestingly, the tumors harboring stop-gained or splice-site mutations in *TP53* displayed significant decreased expression levels, whereas the tumors with other mutation types had significantly increased levels, compared to those of the wild-type ones (Fig 4C). This observation is consistent with our previous study that *TP53* hotspot missense mutations are associated with higher *TP53* RNA and protein expression [33]. We also observed similar effects in the *VHL* mutations in KIRC (S9 Fig). These results suggest that stop-gained and splice-site mutations might act as cis-regulatory drivers of their host genes.

### rDriver enhanced the detection of low frequency driver mutations

To assess whether rDriver can improve the prediction of the low frequency, so-called “tail” mutations, we compared the prevalence of low frequency mutations in our prediction. We found that despite having comparable proportions of low frequency mutations in the driver and the passenger groups (S10 Fig), a significantly bigger portion of the low frequency mutations in the driver group occurred in the CGC genes (S11 Fig) than that in the passenger group. In particular, the doubleton mutations significantly occur to CGC genes in the driver group across all cancer type except LUAD (p<0.05, Fisher’s exact test) This result indicated that rDriver delineated mutations based on their potential functionalities (rather than frequencies). Even more striking functional delineation was observed in mutations of higher frequencies.

### Experimental validation of rDriver predictions using engineered cell-line models

We further evaluated rDriver predictions using an *in vitro* cell growth and transformation assay based on BA/F3 and MCF10A cell-lines [34]. In this functional experiment, a mutant allele was classified as a driver if it appeared activating, i.e., resulted in excessive cell proliferation than the wild-type alleles. It is difficult to obtain a comprehensive and unbiased evaluation due to sparsity of functional validation data. For that reason, we focused on *PIK3CA*, which has been sufficiently validated: 19 of the 22 *PIK3CA* mutations had experimental validation data in our study (S4 Table). Among the 17 mutations that were predicted as drivers by rDriver, 16 appeared activating and 1 had no effect, corresponding to a positive predictive value (PPV) of 0.94. Of the 2 mutations that were predicted as passengers, 1 appeared activating and 1 had no effect, corresponding to a negative predictive value (NPV) of 50%. Eight (8) of the 16 validated driver mutations were potentially novel: not present as actionable mutations in personalizedtherapy.org. Nine (9) of the 16 validated drivers were annotated as non-functional variants by either SIFT or PolyPhen. This experimental validation result, although limited in scope to a single gene, underscores the potential value of rDriver for accurate discovery of novel driver mutations.

### Prognostic association of combined mutation and expression in rDriver prediction

We further assessed the value of rDriver in prognosis. For each patient, we computed an integrative prognostic score (IPS) by multiplying the patient’s mutation, cancer-type-specific association matrix and gene expression data. Based on the distribution of the IPS, we stratified patients in a cancer type into 2 or more subtypes (Methods). For comparison, we also stratified patients based on the expression levels of a set of 500 genes that had the most variable expression levels in the cancer type using negative matrix factorization (NMF) consensus clustering [35].

**Fig 5:**
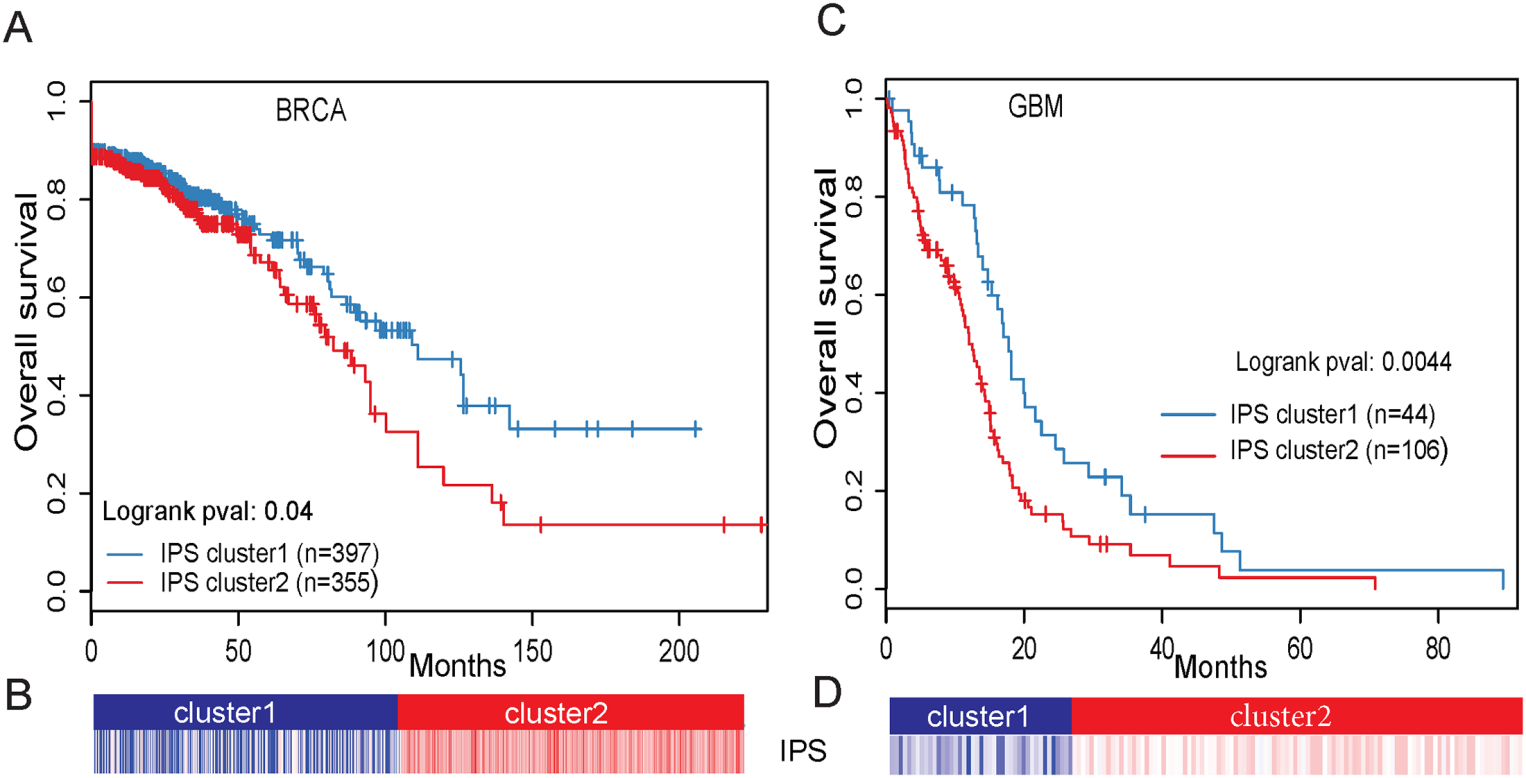
Integrative prognostic scores in two cancer types. (A) Kaplan-Meier plot showing that IPS can significantly separate the tumors in terms of overall survival in BRCA, log-rank test p = 0.041. (B) BRCA samples are classified into 2 clusters based on their IPS values. (C) Kaplan-Meier plot showing that IPS can significantly separate the tumors in terms of overall survival in GBM, log-rank test p = 0.0044. (D) GBM samples are classified into 2 clusters based on their IPS values.

In BRCA, the IPS led to two subtypes with distinct clinical outcome in terms of overall survival (log-rank test p = 0.04) (Fig 5A and 5B), whereas the expression-only analysis resulted in less significant difference (log-rank test p = 0.35) (S12 Fig). Patients in the IPS subtype 2 had a much worse prognosis (n = 355, median survival time of 84.5 months) than those in the IPS subtype 1 (n = 397, median survival time of 114.0 months). A detailed analysis showed that patients in the IPS subtype 1 contained significantly (Student’s T test, p-value = 3.5e-09) higher proportions of *PIK3CA* mutant alleles (e.g., H1047R, E545K, H1047L, and Q546K) than those in the IPS subtype 2 (S13 Fig). In particular, patients with two *PIK3CA* mutation alleles were highly enriched in subtype 1 (Chi-squared test, p-value = 0.1) and all of them were alive before censoring, indicating the prognostic value of the *PIK3CA* mutations. In GBM, the IPS approach was also able to stratify patients into 2 subtypes with distinct survival (log-rank test p-value = 0.0044) with the outcome of subtype 1 (n = 44, median survival time of 17.7 months) being significantly higher than that of the subtype 2 (n = 106, median survival time of 12.0 months) (Fig 5C and 5D). We also found that *IDH1* mutations were significantly enriched in the subtype 1 (Chi-squared test, p-value = 1.4e-05), which was consistent with previous studies [36]. In comparison, stratification based directly on *IDH1* mutation status resulted in less significant outcome (log-rank test, p-value = 0.0058) (S14 Fig) and stratification based only on gene expressions resulted in no significant differences. We also found significant survival stratification based on the IPS in HNSC, SKCM (S5 Table), although the P values were less significant than those obtained from gene expression only analysis.

## Discussion

Accurate identification of driver mutations in specific disease contexts is critical for understanding cancer biology and for developing targeted treatments in the era of precision medicine. However, predicting function based on mutational frequency alone has been challenging due to dramatic genetic heterogeneity among cancer patients, which often results in a large number of low frequency mutations. Previous driver prediction studies have been limited to analyzing mutation data without sufficiently accounting for transcriptional or translational effects of mutations at a population level. As multi-omic data from comprehensive cancer genome sequencing projects become readily available, simultaneous interrogation of genomic, transcriptomic and proteomic data from the same tumor samples may emerge as a powerful approach to identify context-specific driver mutations. In this study, we present a novel approach rDriver, which integrates mutations, functional genomic impact scores and gene expressions to identify driver mutations. It results in increased accuracy compared to existing methods that do not perform such integration. Our work illustrates a principle way to enhance the functionalization of somatic mutations and can potentially accelerate the discovery of novel driver mutations.

rDriver currently utilizes cancer-type specific gene expression and is capable of identifying context-specific or tumor lineage associated drivers. In contrast, the previous mutational hotspot detection method considers only mutational rate variation [37] and may have missed low frequency mutations with systemic regulatory effects. Indeed, through integrative analysis, rDriver predicted not only 81% (129/159) of the previously identified hotspots [37], but also more driver mutations in specific tumor lineages. As a result, the potential cause of 74% of patients can be explained by the rDriver results, as contrast to 54.8% by hotspot mutations only. We also demonstrated that the integrative prognostic scores computed using rDriver could lead to more accurately prognosis of the BRCA and the GBM patients. These results provide a rationale for further investigating the utilities of rDriver in cancer target discovery and development.

It is interesting to note that rDriver assumed that all regulated genes are independent and did not take advantage the prior pathway information or known gene regulatory network. This caveat may limit its power to predict the driver mutations that enable the regulatory function in a specific pathway. At same time, the context free prediction of rDriver lends itself to recover the driver that has regulatory effects unaccounted for by the known interaction network. For example, a known network-based approaches such as xSeq did not report *PIK3CA* as a top candidate driver gene in breast cancer [18]. In contrast, rDriver, which examines the expression levels of all the genes (including those in distant pathways), indicated *PIK3CA* as a driver gene that impacts cell cycle, amino acid metabolism, cancer pathway, etc.

Our study has several caveats that will need to be addressed in the future.

First, the set of mutations that we included in our study are primarily protein coding mutations detected via whole exome sequencing. This is not a principle limitation of our method but reflects a limitation of publically available resource. In principle, the rDriver framework can potentially include both coding and non-coding mutations, although more samples will likely be needed when non-coding mutations are included. It is our expectation that rDriver will be able to improve the prediction of non-coding driver mutations through integration of gene expression or other quantitative trait data, as having been demonstrated in previous genome-wide eQTL studies [38]. The novel statistical framework of rDriver makes it possible to systematically integrate additional functional genomic information such as protein-DNA interaction, DNA methylation, chromatin modification, etc., which have shown to be useful for predicting non-coding driver mutations [39].

Second, we only included SNVs and indels in our investigation but did not include copy number alteration (CNAs) that are predictive of gene expressions [38, 40]. That was because the functional genomic properties (i.e., the FIS) of CNAs and other segmental events are fundamentally different from those of SNVs and indels. Including them with SNVs and indels will require further investigation beyond the scope of this study. Despite this known caveat, our algorithm was able to explain a considerable fraction of gene expression variances and transform them into the prediction of driver mutations.

Third, similar to other association studies, results obtained from rDriver are likely confounded by additional genetic, epigenetic, pathological (e.g., intratumor heterogeneity), tissue or environmental factors. To assess potential biases, we processed the raw gene expression data using log transformation and quantile normalization, followed by a principal component analysis. We did not find evident population stratification that may systematically bias driver mutation discovery (S15 Fig). We will further investigate and deconvolute these potential confounding factors in our future studies.

Finally, although we examined only mRNA expression data in this study, rDriver can potentially be generalized to integrate other types of molecular data such as protein and phosphoprotein activity levels produced by reverse phase protein arrays [41] and splicing alteration [42]. These additional data types will likely provide fuels to identify new drivers.

In summary, the results from our study indicated that our novel method rDriver has successfully transformed the power of multi-omic datasets into more accurate driver mutation prediction in individual patients. An important highlight in our study is that we used engineered cell-line models to validate a subset of our computational predictions. Although limited in scales and by potential bias of the cell-line models, the experimental validation results provided independent measurement that confirmed the accuracy of rDriver in driver mutation discovery. Overall, the results were encouraging and could constitute a solid step towards solving one of the most pressing challenges in cancer genomics and personalized medicine.

## Methods

### The rDriver model

Consider two separate data profiles with G gene expression levels and M mutations in *N* samples. Let $y_{j}=[y_{j_{1},}y_{j_{2}},\ldots {,y}_{j_{N},}]$ and $x_{k}=[x_{k_{1},}x_{k_{2}},\ldots {,x}_{k_{N},}]$ be the expression levels of gene j and mutation status of allele k, in samples 1 to N. A linear relationship between the expression of gene j and the mutation k is assumed and represented by the following linear model,

$${\text{y}}_{\text{j}}=\sum_{\text{k}=1}^{\text{M}}\beta_{\text{jk}}.{\text{x}}_{\text{k}}+\varepsilon_{\text{j}},$$ (1)

where ε_j_ represents a standard Gaussian noise, and β_jk_ representing the amount of association for each mutation-gene pair. We further assume that x_j_ are standardized and consider a model without intercept. Within this context, the goal of study is to find, for each gene, a small subset of mutations that affect its expression level. Usually, the number of candidate mutations is larger than the number of samples, which results in a high-dimensional model selection problem. To ensure proper estimation of parameter β, a L_1_ and L_2_ penalty was added to the least square estimator (known as the LASSO) [43]. Given the sparse nature of the associations, most of the regression coefficients shrink to 0 and the nonzero ones will be recovered. To incorporate the prior functional impact scores (FISs), we introduce a scaling parameter δ to regularize the estimation of β. We define δ_jk_ as a function of FISs that affect the pair interaction between the j^*th*^ gene and the k^*th*^ mutation:

$$\delta_{\text{jk}}=\text{sigmoid}\left(\sum_{i=1}^{F}w_{i}f_{i}\right)=\frac{1}{1+\text{exp}(-\sum_{i=1}^{F}w_{i}f_{i})}$$ (2)

where *f*_*i*_ is the *i*^th^ FIS (or feature) among total F ones that characterizes the potential impact of mutation k on the expression of gene j. Since we are interested in the relative contributions from different FISs, we further weigh them by *w*_*i*_ ≥ 0.

Therefore, an L_1_ and an additional L_2_ norm place constraints on the estimation of the coefficients and feature weights respectively with adaptive scaling parameter is enforced in the model as follows:

$${\text{min}}_{\beta }\left\{\|{\text{y}}_{\text{j}}-\sum_{\text{k}=1}^{\text{K}}\beta_{\text{jk}}.{\text{x}}_{\text{k}}{\|}_{2}+\lambda_{1\text{j}}\sum_{\text{k}=1}^{\text{K}}\left|\delta_{\text{jk}}.\beta_{\text{jk}}\right|+\lambda_{2\text{j}}\sum_{\text{k}=1}^{\text{M}}\left(\sum_{i=1}^{F}w_{ki}^{2}\right)\right\}$$ (3)

where λ_1j_ and λ_2j_ determines the degree of regularization of β_jk_ and *w*_*ki*_ through L_1_ and L_2_ respectively. This is a convex optimization problem and thus, if an algorithm is able to find a local minimum, it can find the global minimum.

rDriver uses an iterative coordinate descent algorithm to minimize the above objective function (Fig 1). The iteration alternates between two steps. In one step, we optimize over *w*’s given the current λ_1_, and λ_2_, and in the other step we optimize over λ_1_, and λ_2_ given the current *w*’s. The regularization parameters λ_1_, and λ_2_ are determined through a 10-fold cross validation procedure.

To obtain the p-value for each mutation, we first run rDriver for each gene and obtain a solution matrix representing the paired associations between genes and mutations. We then summed up the number of genes associated with a specific mutation by nonzero regression coefficients. We calculated the null distribution based on permutations of the original gene expression matrix followed by running rDriver with identical parameters. After a large number of permutations, we calculated empirical p-values based on the percentiles of the observed numbers of associations in respective null distributions.

### Gene expression data, mutation and its clone status data collection

The data (https://www.synapse.org/#!Synapse:syn300013) include mutations and expression data of 8 TCGA cancer types. Differential gene expression (DEG) analysis was performed using Mann-Whitney U-test for each mutation. The obtained p-values were further corrected by Benjamini and Hochberg (BH) multiple testing procedure, and the False Discovery Rate (FDR) <0.05 was considered as significantly differential expression gene. In preprocessing step of rDriver analysis, we first applied log2 transformation of mRNA RPKM expression values followed by further quantile normalization across all patients and then screened the genes for cancer related targeting genes (~3,030) in eQTL mapping. The clonal status and cancer cell fraction were downloaded from https://bitbucket.org/nmcgranahan/pancancerclonality/downloads.

### Frequency based driver prediction

We predicted drivers based only on mutational frequency. At gene level, drivers were determined based on the number of times a gene was mutated in the cohort, regardless of the type, position and amino acid alteration of individual mutations. At mutation level, drivers were determined based on the number of times a mutation was observed in the cohort. A fixed cutoff on the number of occurrence divided the set of genes or mutations into drivers and passengers.

### Mutation annotation

We annotated single nucleotide variants (SNVs) and small indels using Annovar [44], GERP, SIFT [20] and other programs and compared them with dbSNP, COSMIC [45], and TCGA databases, in order to understand their potential functional consequence. For the GERP scores, we extracted the resistant substitution (RS) scores from the nucleotide bases that belong to each candidate mutation. A higher RS score represents stronger evolutionary conservation.

### KEGG enrichment analysis

We extracted the KEGG pathway annotations from KEGG database. Among total 284 pathways, we examined the enrichment for the associated gene list. For a specific mutation’s associated genes, we search for pathway that are significantly over-represented compared to the original 3030 genes. The enrichment p-values were calculated by the hyper-geometric test. The obtained p-values were further corrected by Benjamini and Hochberg (BH) multiple testing procedure, and the False Discovery Rate (FDR) <0.05 was considered as significantly differential expression gene. The pathway terms with adjusted p-values < 0.05 were considered as significantly enriched by the targeted gene list.

### Functional test of the mutations

Mutation clones were first constructed using High-Throughput Mutagenesis and Molecular Barcoding (HiTMMoB) technology [34]. Stable cell lines including IL-3-dependent Ba/F3 cells and EGF- and insulin-dependent MCF10A cells were then established expressing *PIK3CA* variants. The mutations are introduced into IL-3-dependent Ba/F3 cells and EGF- and insulin-dependent MCF10A cells using lentiviral approach. After spinoculation, cells were incubated with medium without the dependent growth factors for 3 weeks. Cell viability of transduced cells was examined by CellTiter-Glo assay (Promega) at 1, 1.5, 2 and 3 weeks post-transduction time points. The level of activity was assigned based on the level of increased cell viability of mutant comparing to wild-type.

### Tumor clustering and survival analysis

We used different features associated with each patient to classify the tumor samples including the 500 genes with most variable expression and mutation data, respectively. Cluster analysis was performed on alternative numbers of clusters (2 to 6) based on NMF consensus clustering. To cluster the integrative prognostic score, we used a one-dimensional K-mean consensus-clustering algorithm. The optimized cluster number is selected based on cophenetic correlation [35]. We used a log-rank test to examine whether the clusters or mutations significantly correlated with patient overall survival time (available at https://tcga-data.nci.nih.gov/tcga/) and used Kaplan-Meier plot to demonstrate survival distributions.

### Software availability

The source code of rDriver is available at http://bioinformatics.mdanderson.org/main/RDriver.

## Supporting information

S1 Dataset The names of 3,030 genes. (XLSX)

S2 Dataset The mutation list ordered based on the p-values obtained from rDriver. (XLSX)

S3 Dataset KEGG enrichment analysis for the associated genes of mutations in BRCA. (XLSX)

S4 Dataset The benchmark set of cancer driver genes and driver mutations for ROC analysis including 76 highly possible driver genes from CGC, 125 genes from Volgestein et al. 2013, and 877 driver mutations from Martelotto, Ng et al. 2014. (XLSX)

S5 Dataset The 1389 predicted driver across 8 cancer types by rDriver. (XLSX)

S1 Fig Histogram of the proportion of variance explained by the selected mutations for each mRNA expression in BRCA. (PDF)

S2 Fig The DEG analysis. **(A)** The top 9 most frequent mutations and their gene DEG analysis in BRCA. The grey bar represents the mutant frequency and red line denotes the number of differentially expressed genes associated with mutations. (B) The rank correlation between rDriver score and the number of DEG associated is 0.82 with p value equalt to 0.002 based on the top 9 most frequent mutations.
(PDF)

S3 Fig The Heatmap of association matrix between the ten most abundant KEGG pathways and the 20 highest scoring mutations predicted by rDriver. Colors represent the extent of associations between mutations and pathways, obtained from gene enrichment analysis. The bar-plot on the right shows the counts of significant KEGG pathways associated with the mutations.
(PDF)

S4 Fig ROC curves comparing the set of the driver genes predicted by various programs against a set of 125 known cancer driver genes in from Vogelstein et al., 2013. (PDF)

S5 Fig Summery of rDriver prediction across 8 cancer types. (**A**) the distribution of genes with varied mutant alleles. (**B**) The distribution of mutant alleles with varied tumor types.
(PDF)

S6 Fig ROC analysis with 7 cancer types. The cancer type is indicated at the top. The number of high possible cancer genes in each cancer type is indicated at the bottom.
(PDF)

S7 Fig The cancer cell fraction of mutations in driver and passenger groups within each cancer types. Significance (Stars) from Wilcox test is indicated. Exact p values are as follows; BLCA, p = 0.003; BRCA, p = 0.00001; GBM, p = 0.0001; KIRC, p = 0.0001; HNSC, p = 0.011; LUDA, p = 0.017; LUSC, p = 0.045; SKCM, p = 0.014.
(PDF)

S8 Fig Probability distributions over the cancer cell fraction for co-mutated drivers in specific tumors. (A) The *PIK3CA* E545K and EGFR A224V in GBM. (B) The *PIK3CA* E545K and VHL Q145* in KIRC.
(PDF)

S9 Fig Altered VHL gene expression associated with different types of mutations in KIRC. The t test does not show any significance.
(PDF)

S10 Fig Frequency distribution between the driver and the passenger groups. Stars indicate significance of enrichment of the related frequency in driver group. (**<0.01, *p<0.05; Fisher’s exact test).
(PDF)

S11 Fig The proportion of mutations that occur to CGC is different between the two groups. Stars indicate significance of enrichment of the related frequency occurring in CGC in driver group. (**<0.01, *p<0.05; Fisher’s exact test).
(PDF)

S12 Fig Kaplan-Meier plot showing that the genes with max variance has no power to separate the tumors in terms of overall survival in BRCA. Log-rank test p = 0.35.
(PDF)

S13 Fig The prevalence of mutations for the top 20 significant drivers across 2 clusters in BRCA. (PDF)

S14 Fig Kaplan-Meier plot showing that the status of IDH1 R132 mutation has significant power to separate the tumors in terms of overall survival in GBM. Log-rank test p = 0.0058.
(PDF)

S15 Fig Scatter plot of the first two principal components of gene expression of the 3,030 genes across 8 cancer types. (PDF)

S1 Table Data summary of pan cancer 8 including mutation, expression and their patient numbers. (DOCX)

S2 Table The genes carrying novel mutations of rDriver prediction in 8 cancer types, and their function annotation from literatures. (DOCX)

S3 Table The number of clonal or subclonal mutations in driver and passenger group across 8 cancer types. (DOCX)

S4 Table In vitro experimental functional validation of mutations using BA/F3 and MCF10A cell lines. (DOCX)

S5 Table The significance of survival separation of subgroups based on IPS and mRNA expression, respectively. (DOCX)
