# Supplementary material for: Cancer driver mutation prediction through Bayesian integration of multi-omic data
Source: PLoS One. 2018 May 8;13(5):e0196939. doi: 10.1371/journal.pone.0196939 (PMC5940219; doi:10.1371/journal.pone.0196939)
Supplement: S1 Fig — (PDF) [file pone.0196939.s006.pdf]

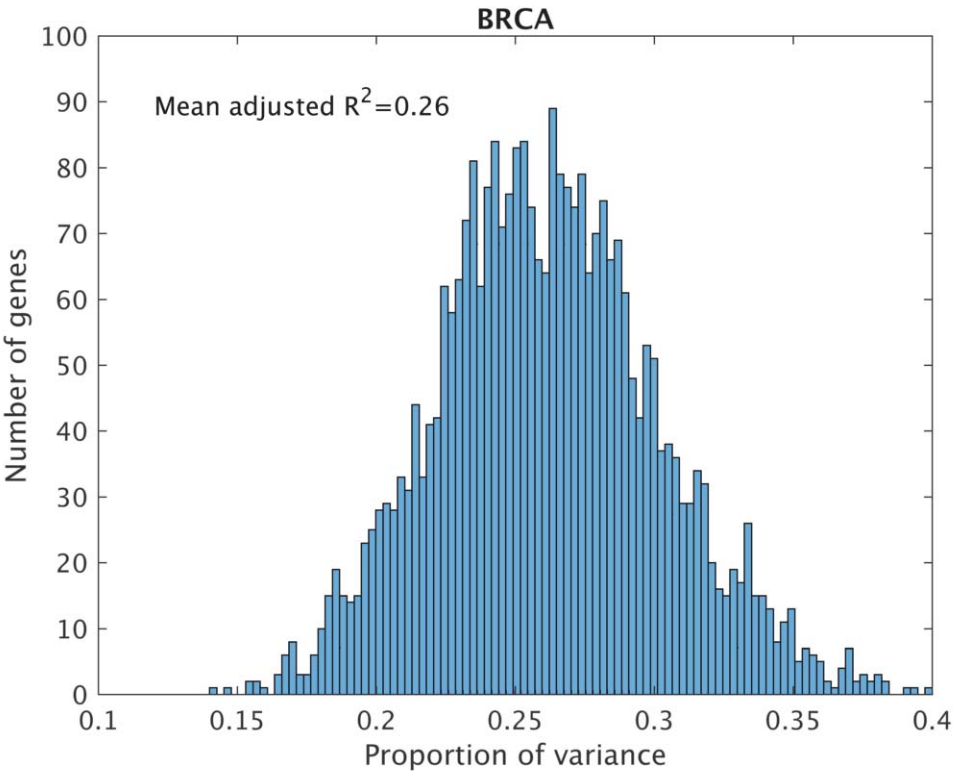

S1 Fig. Histogram of the proportion of variance explained by the selected mutations for each mRNA expression in BRCA
