# Supplementary material for: Cancer driver mutation prediction through Bayesian integration of multi-omic data
Source: PLoS One. 2018 May 8;13(5):e0196939. doi: 10.1371/journal.pone.0196939 (PMC5940219; doi:10.1371/journal.pone.0196939)
Supplement: S2 Fig — (A) The top 9 most frequent mutations and their gene DEG analysis in BRCA. The grey bar represents the mutant frequency and red line denotes the number of differentially expressed genes associated with mutations. (B) The rank correlation between rDriver score and the number of DEG associated is 0.82 with p value equalt to 0.002 based on the top 9 most frequent mutations. (PDF) [file pone.0196939.s007.pdf]

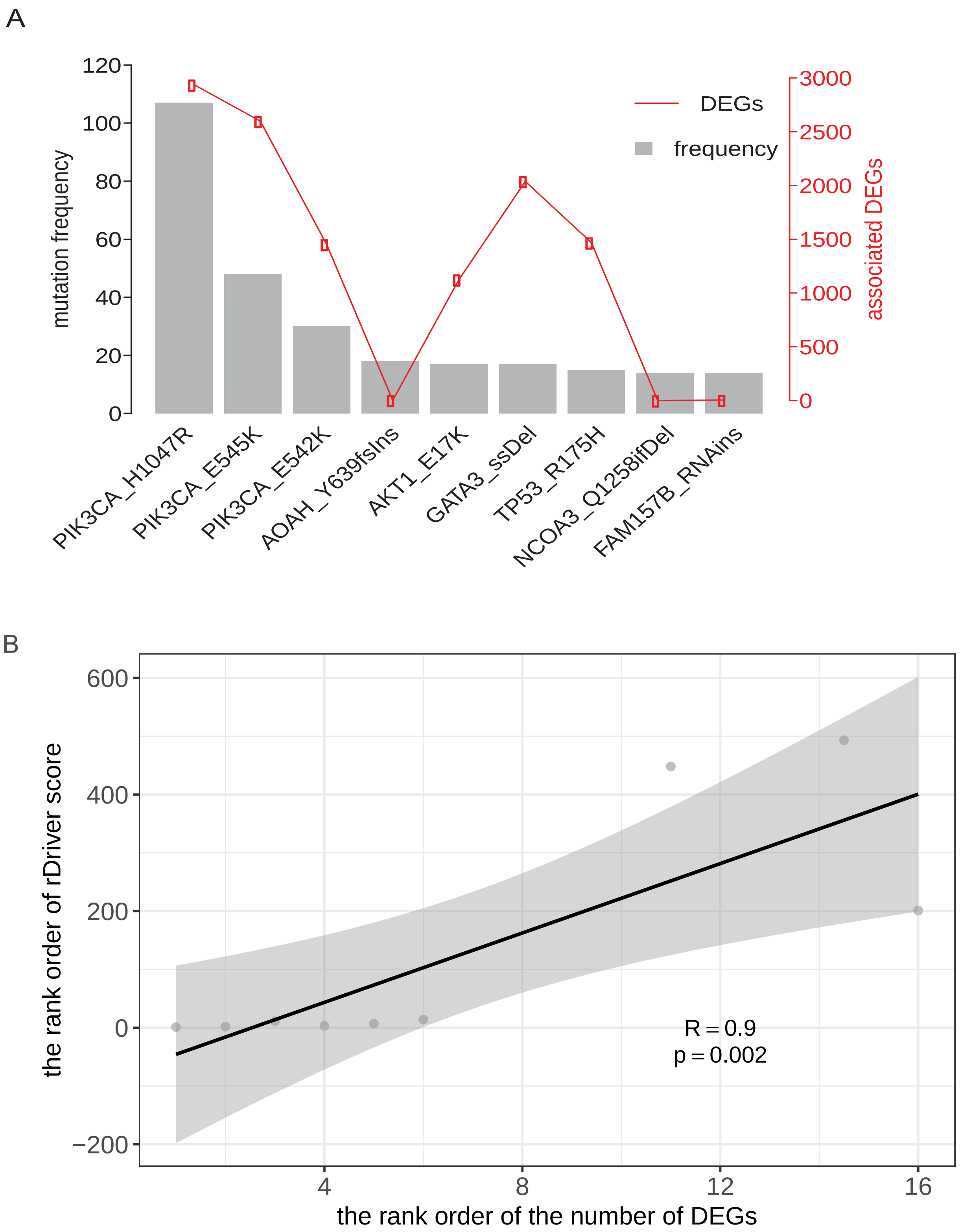

S2 Fig. The DEG analysis (A) The top 9 most frequent mutations and the number of associated DEGs in BRCA. The gray bar and left axis indicate the mutation frequency for the mutations. The red line and right axis denote the number of associated DEGs. (B) The rank correlation between rDriver score and the number of DEG associated is 0.82 with p value equal to 0.002 based on the top 9 most frequent mutations.
