# Supplementary material for: Cancer driver mutation prediction through Bayesian integration of multi-omic data
Source: PLoS One. 2018 May 8;13(5):e0196939. doi: 10.1371/journal.pone.0196939 (PMC5940219; doi:10.1371/journal.pone.0196939)
Supplement: S3 Fig — Colors represent the extent of associations between mutations and pathways, obtained from gene enrichment analysis. The bar-plot on the right shows the counts of significant KEGG pathways associated with the mutations. (PDF) [file pone.0196939.s008.pdf]

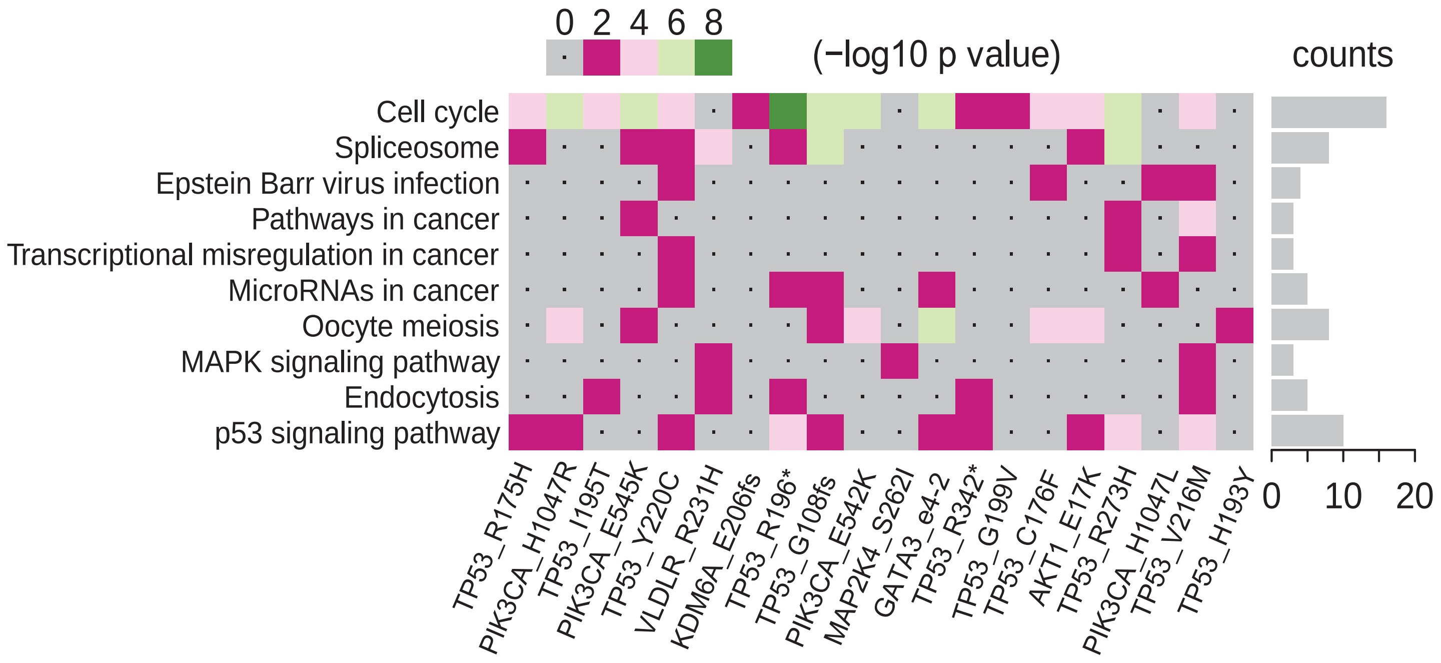

S3 Fig. A Heatmap depicting association between the ten most abundant KEGG pathways and the 20 highest scoring mutations predicted by rDriver. Colors represent the extent of associations between mutations and pathways, obtained from gene enrichment analysis. The bar-plot on the right shows the counts of significant KEGG pathways associated with the mutations.
