# Supplementary material for: Cancer driver mutation prediction through Bayesian integration of multi-omic data
Source: PLoS One. 2018 May 8;13(5):e0196939. doi: 10.1371/journal.pone.0196939 (PMC5940219; doi:10.1371/journal.pone.0196939)
Supplement: S4 Fig — (PDF) [file pone.0196939.s009.pdf]

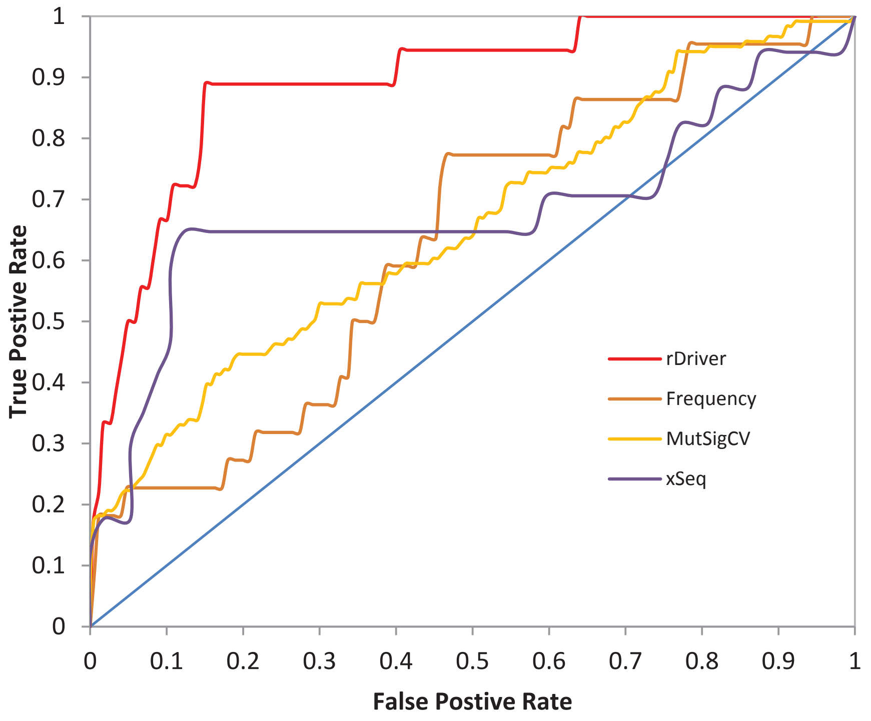

S4 Fig. ROC curves comparing the set of the driver genes predicted by various programs against a set of 125 known cancer driver genes in from Vogelstein et al., 2013
