# Supplementary material for: Cancer driver mutation prediction through Bayesian integration of multi-omic data
Source: PLoS One. 2018 May 8;13(5):e0196939. doi: 10.1371/journal.pone.0196939 (PMC5940219; doi:10.1371/journal.pone.0196939)
Supplement: S5 Fig — (A) the distribution of genes with varied mutant alleles. (B) The distribution of mutant alleles with varied tumor types. (PDF) [file pone.0196939.s010.pdf]

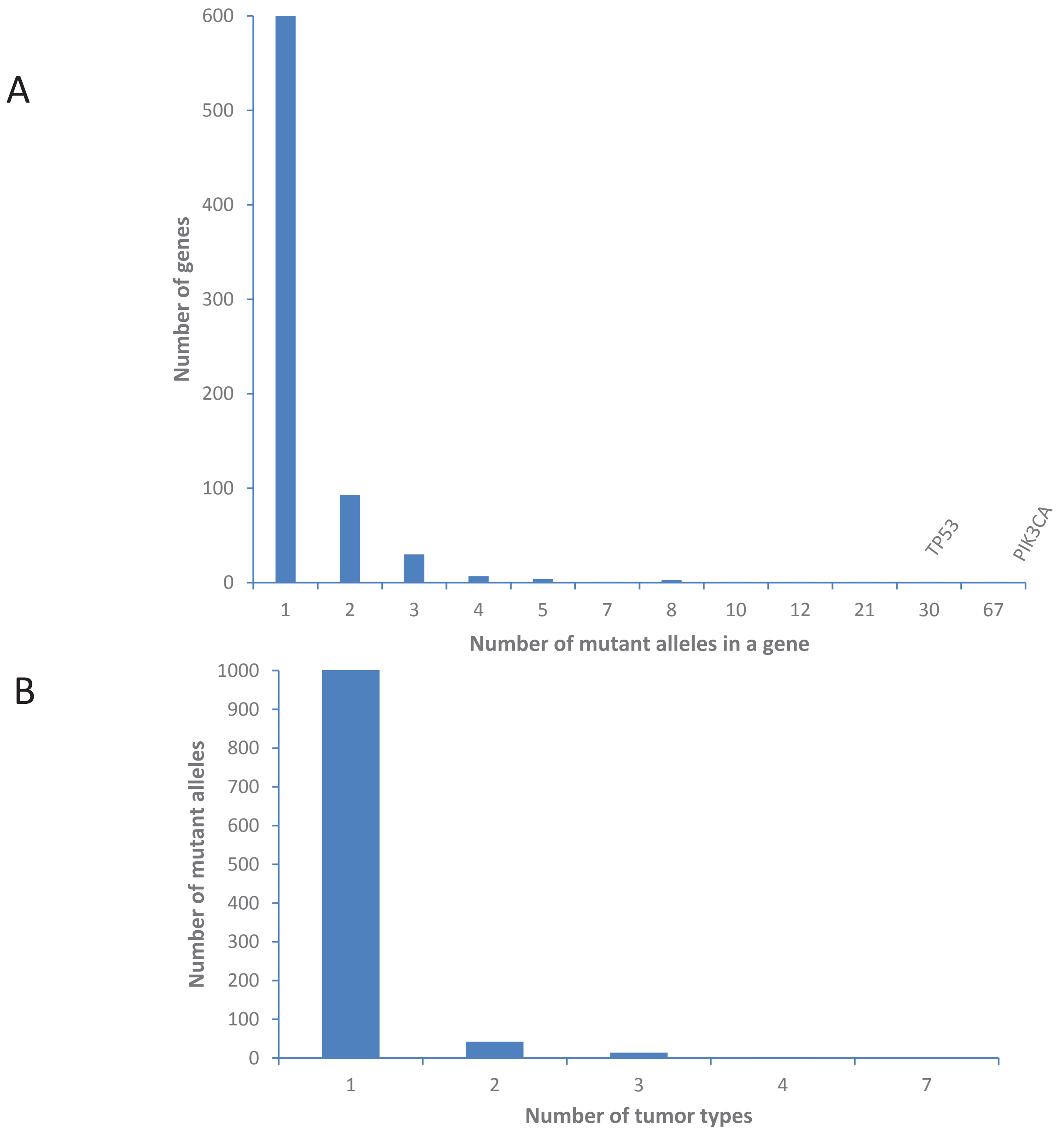

S5 Fig. Summary of rDriver prediction across 8 cancer types. (A) The distribution of genes with varied mutant alleles. The two most populated genes PIK3CA and TP53 are labeled.
(B) The distribution of mutant alleles with varied tumor types.
