# Supplementary material for: Cancer driver mutation prediction through Bayesian integration of multi-omic data
Source: PLoS One. 2018 May 8;13(5):e0196939. doi: 10.1371/journal.pone.0196939 (PMC5940219; doi:10.1371/journal.pone.0196939)
Supplement: S6 Fig — The cancer type is indicated at the top. The number of high possible cancer genes in each cancer type is indicated at the bottom. (PDF) [file pone.0196939.s011.pdf]

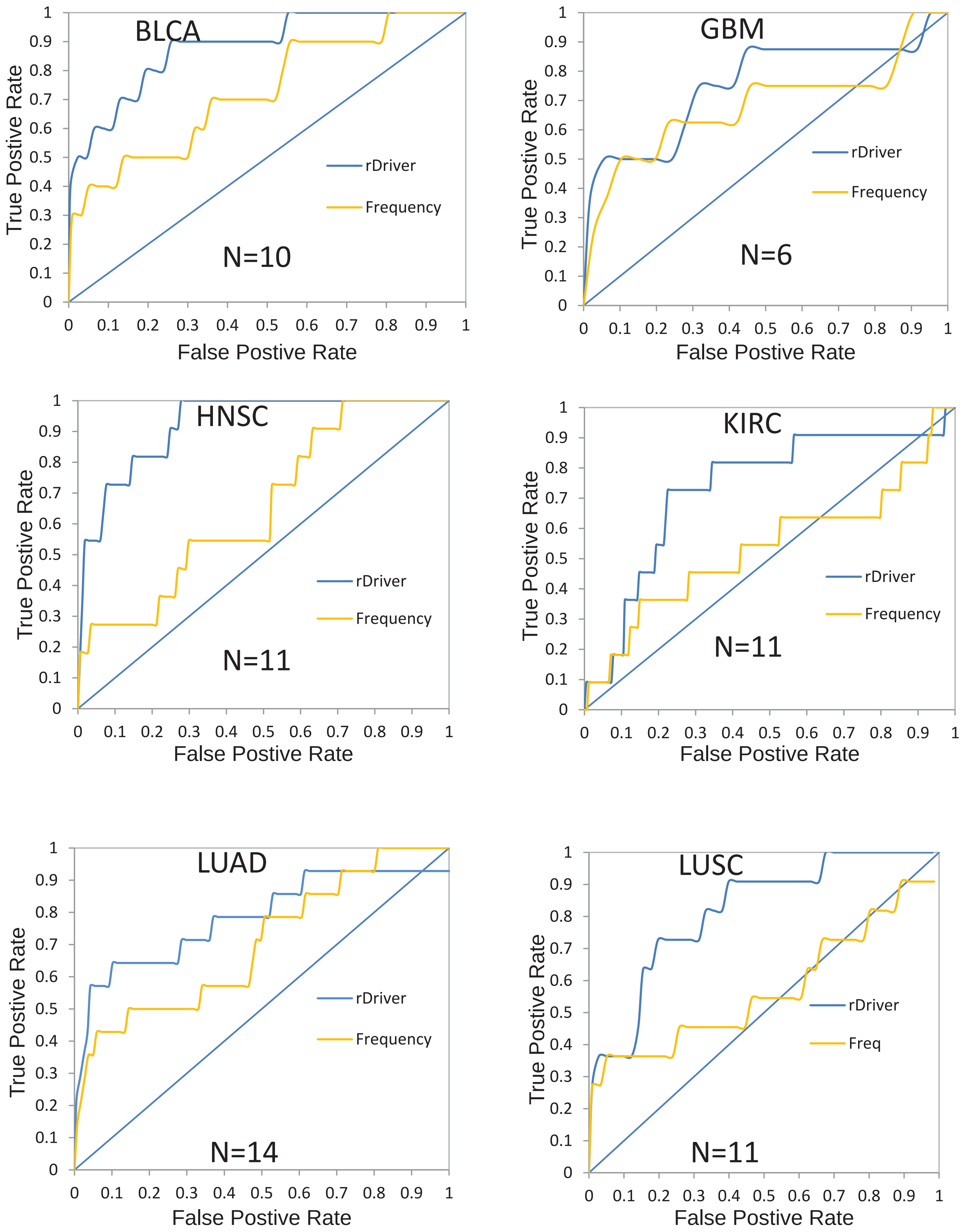
BLCA
rDriver
Frequency
N=10
True Postive Rate
False Postive Rate
GBM
rDriver
Frequency
N=6
True Postive Rate
False Postive Rate
HNSC
rDriver
Frequency
N=11
True Postive Rate
False Postive Rate
KIRC
rDriver
Frequency
N=11
True Postive Rate
False Postive Rate
LUAD
rDriver
Frequency
N=14
True Postive Rate
False Postive Rate
LUSC
rDriver
Freq
N=11
True Postive Rate
False Postive Rate

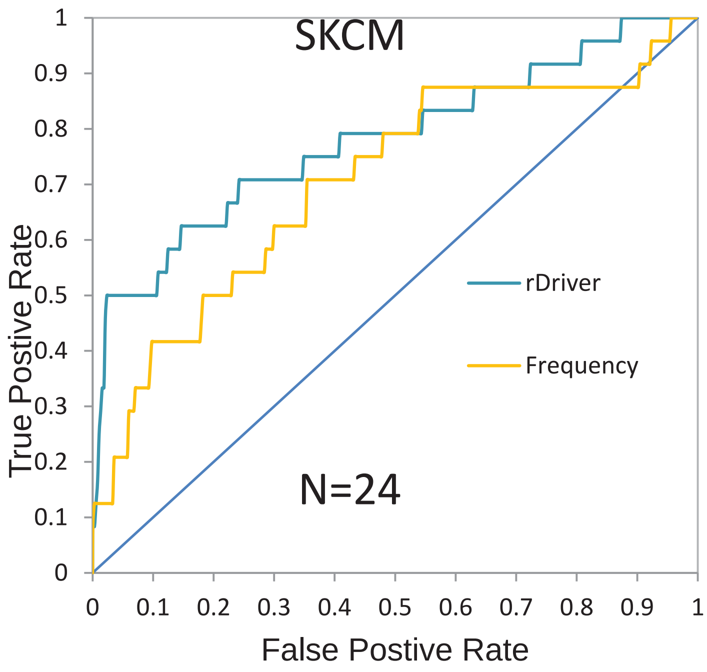

S6 Fig. ROC analysis with 7 cancer types. The cancer type is indicated at the top. The number of high possible cancer genes in each cancer type is indicated at the bottom.
