# Supplementary material for: Cancer driver mutation prediction through Bayesian integration of multi-omic data
Source: PLoS One. 2018 May 8;13(5):e0196939. doi: 10.1371/journal.pone.0196939 (PMC5940219; doi:10.1371/journal.pone.0196939)
Supplement: S7 Fig — Significance (Stars) from Wilcox test is indicated. Exact p values are as follows; BLCA, p = 0.003; BRCA, p = 0.00001; GBM, p = 0.0001; KIRC, p = 0.0001; HNSC, p = 0.011; LUDA, p = 0.017; LUSC, p = 0.045; SKCM, p = 0.014. (PDF) [file pone.0196939.s012.pdf]

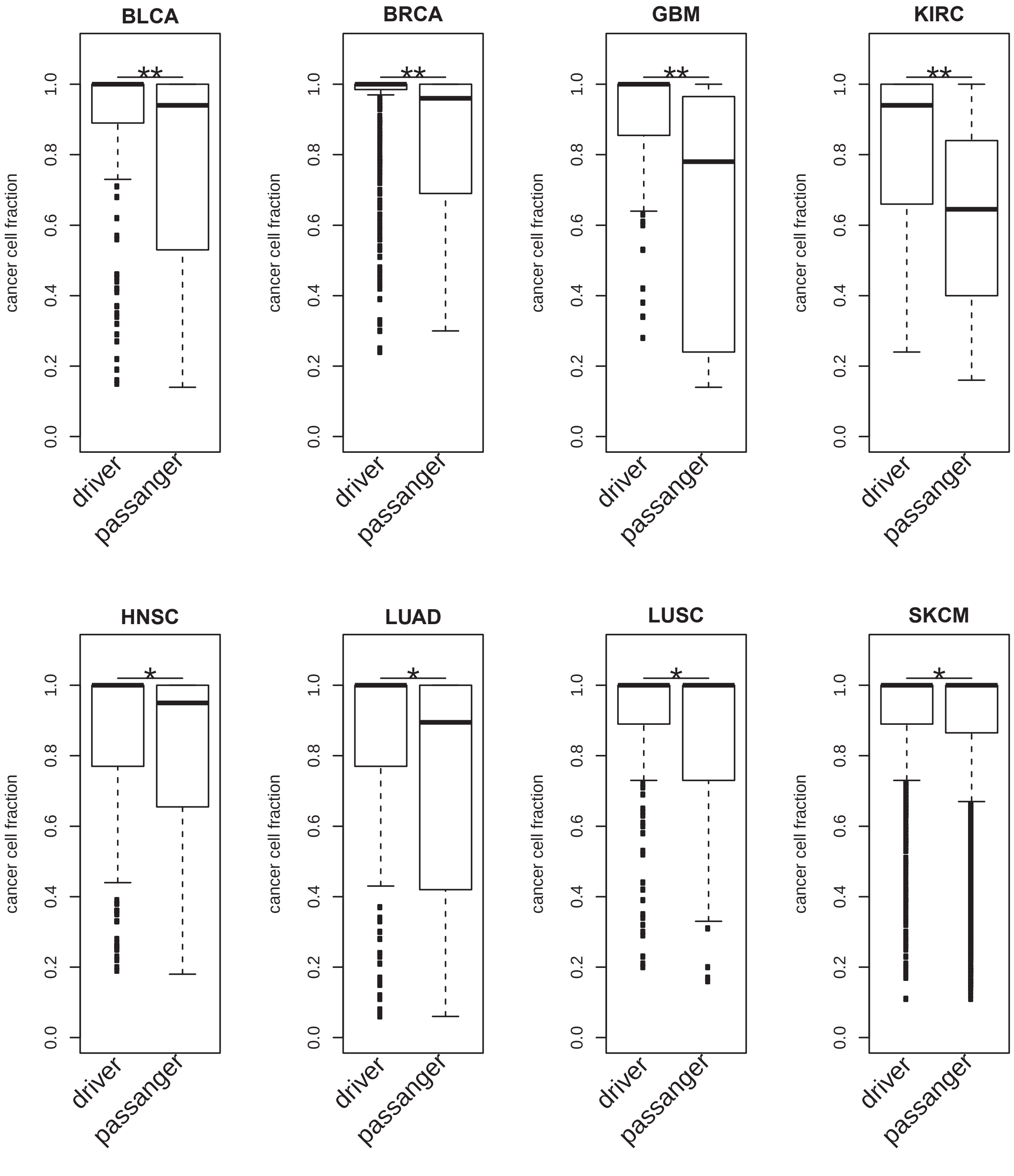

S7 Fig. The cancer cell fraction of mutations in driver and passenger groups within each cancer types. Significance (Stars) from Wilcox test is indicated. Exact p values are as follows; BLCA, p=0.003; BRCA, p=0.00001; GBM, p=0.0001; KIRC, p=0.0001; HNSC, p=0.011; LUDA, p=0.017; LUSC, p=0.045; SKCM, p=0.014.
