# Supplementary material for: Cancer driver mutation prediction through Bayesian integration of multi-omic data
Source: PLoS One. 2018 May 8;13(5):e0196939. doi: 10.1371/journal.pone.0196939 (PMC5940219; doi:10.1371/journal.pone.0196939)
Supplement: S8 Fig — (A) The PIK3CA E545K and EGFR A224V in GBM. (B) The PIK3CA E545K and VHL Q145* in KIRC. (PDF) [file pone.0196939.s013.pdf]

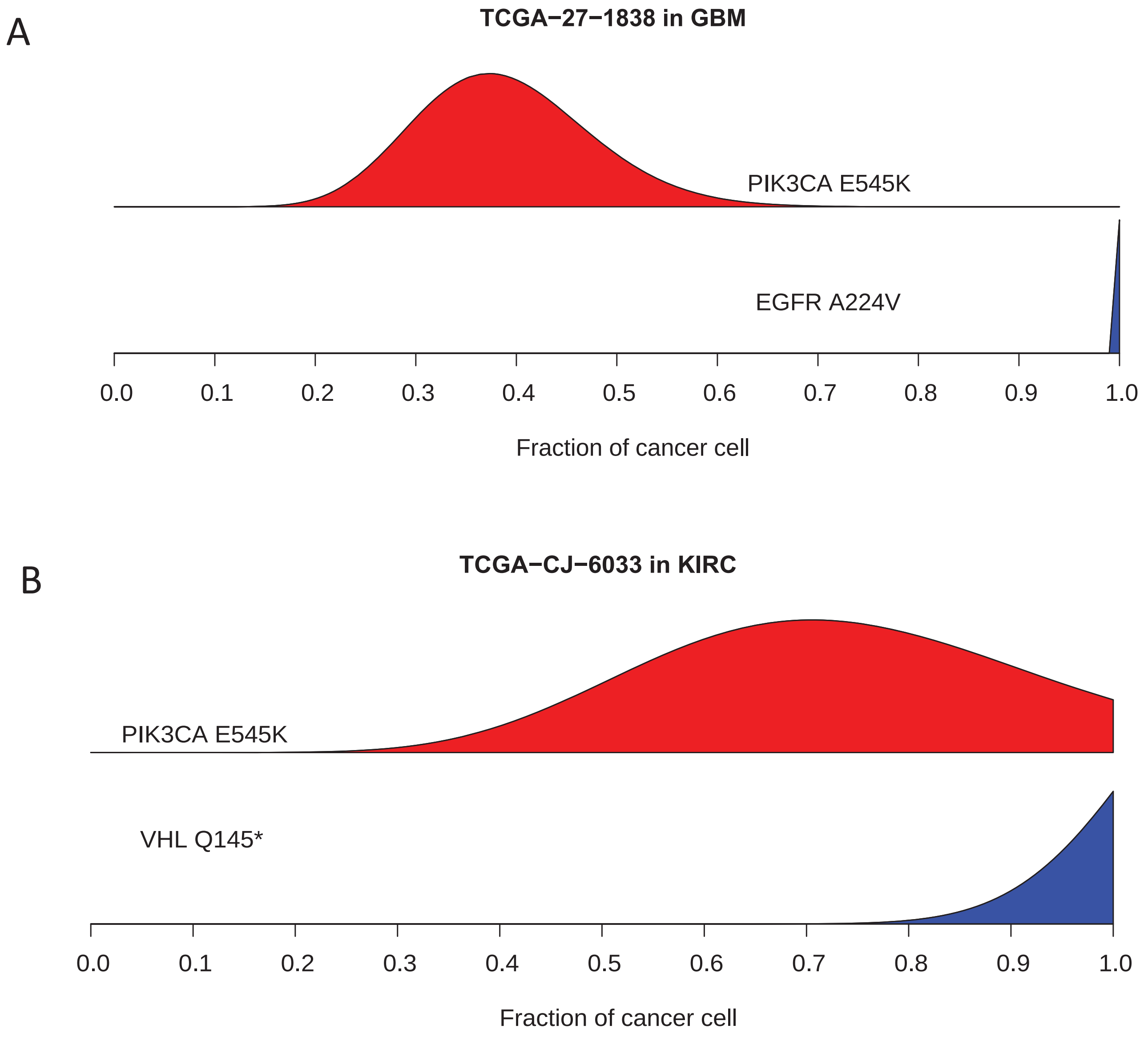

S8 Fig. Probability distributions over the cancer cell fraction for co-mutated drivers in specific tumors. (A) The PIK3CA E545K and EGFR A224V in GBM. (B) The PIK3CA E545K and VHL Q145* in KIRC.
