# Supplementary material for: Cancer driver mutation prediction through Bayesian integration of multi-omic data
Source: PLoS One. 2018 May 8;13(5):e0196939. doi: 10.1371/journal.pone.0196939 (PMC5940219; doi:10.1371/journal.pone.0196939)
Supplement: S9 Fig — The t test does not show any significance. (PDF) [file pone.0196939.s014.pdf]

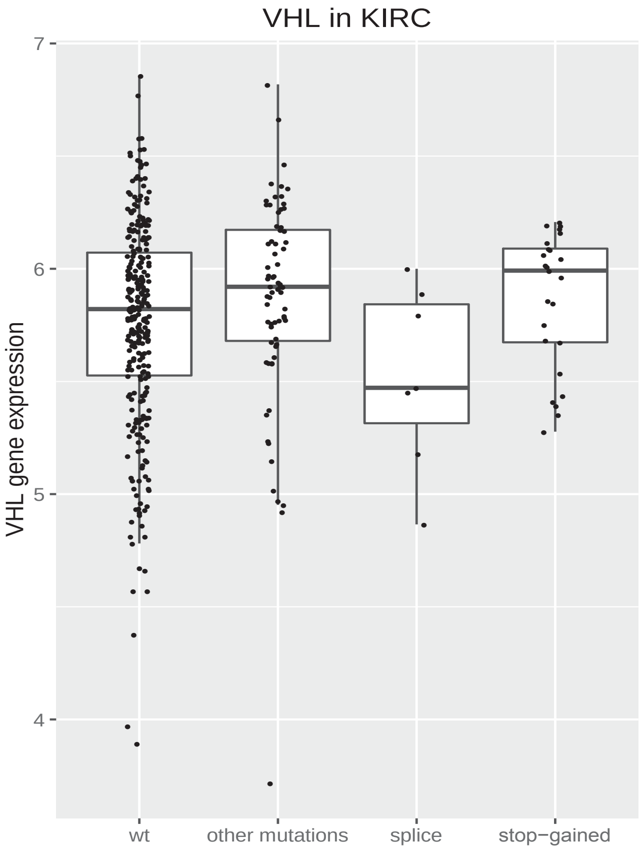

S9 Fig. The altered VHL gene expression associated with different types of mutations in KIRC, The t test does not show any significance.
