# Supplementary material for: Cancer driver mutation prediction through Bayesian integration of multi-omic data
Source: PLoS One. 2018 May 8;13(5):e0196939. doi: 10.1371/journal.pone.0196939 (PMC5940219; doi:10.1371/journal.pone.0196939)
Supplement: S10 Fig — Stars indicate significance of enrichment of the related frequency in driver group. (**<0.01, *p<0.05; Fisher’s exact test). (PDF) [file pone.0196939.s015.pdf]

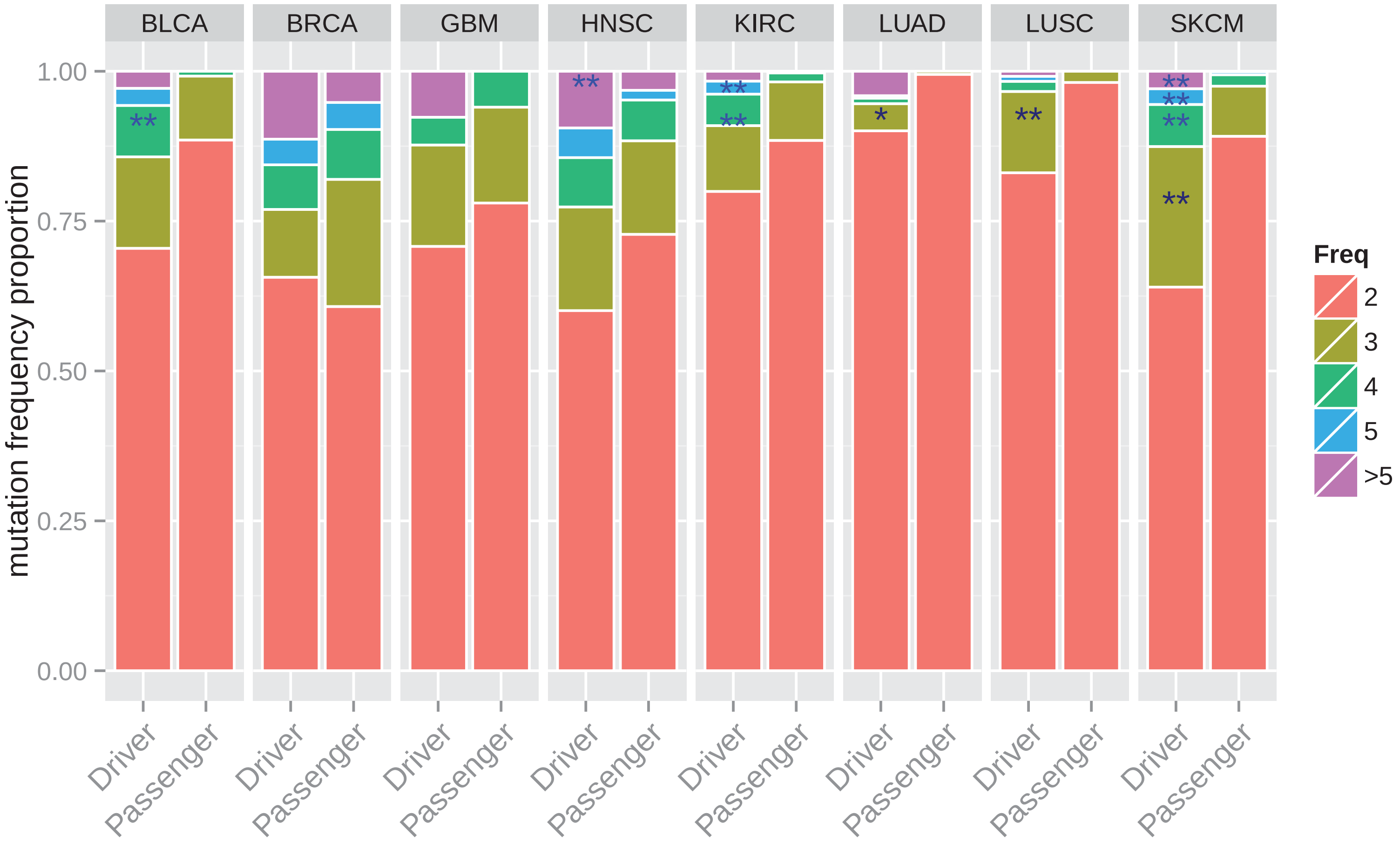

S10 Fig. Frequency distribution between the driver and the passenger groups. Stars indicate significance of enrichment of the related frequency in driver group. (**<0.01, *p<0.05; Fisher's exact test).
