# Supplementary material for: Cancer driver mutation prediction through Bayesian integration of multi-omic data
Source: PLoS One. 2018 May 8;13(5):e0196939. doi: 10.1371/journal.pone.0196939 (PMC5940219; doi:10.1371/journal.pone.0196939)
Supplement: S12 Fig — Log-rank test p = 0.35. (PDF) [file pone.0196939.s017.pdf]

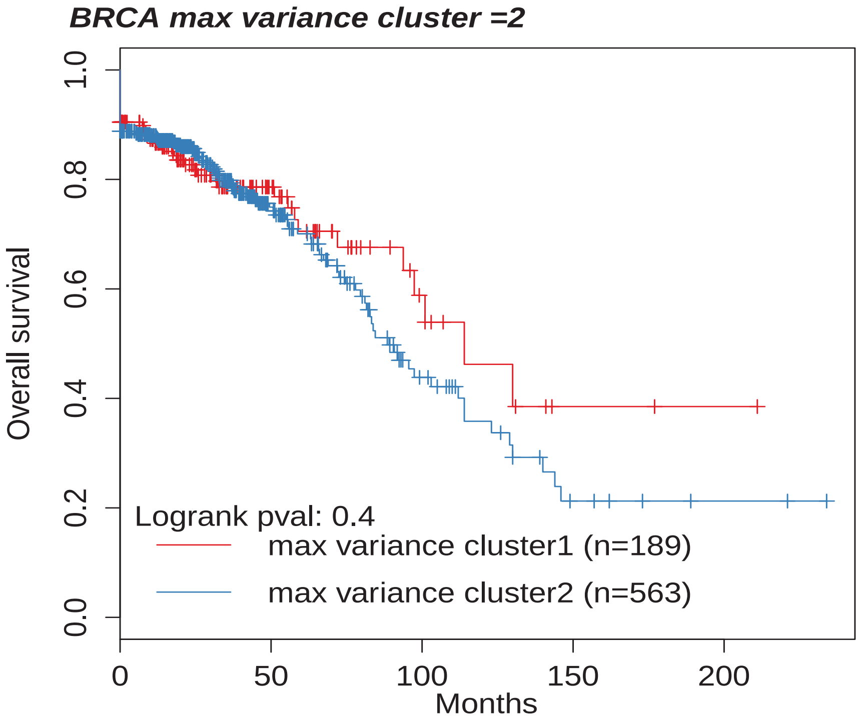

S12 Fig. Kaplan-Meier plot showing that the genes with max variance has no power to separate the tumors in terms of overall survival in BRCA, log-rank test p=0.35.
