# Supplementary figures and images for: Cancer driver mutation prediction through Bayesian integration of multi-omic data
Source: PLoS One. 2018 May 8;13(5):e0196939. doi: 10.1371/journal.pone.0196939 (PMC5940219; doi:10.1371/journal.pone.0196939)

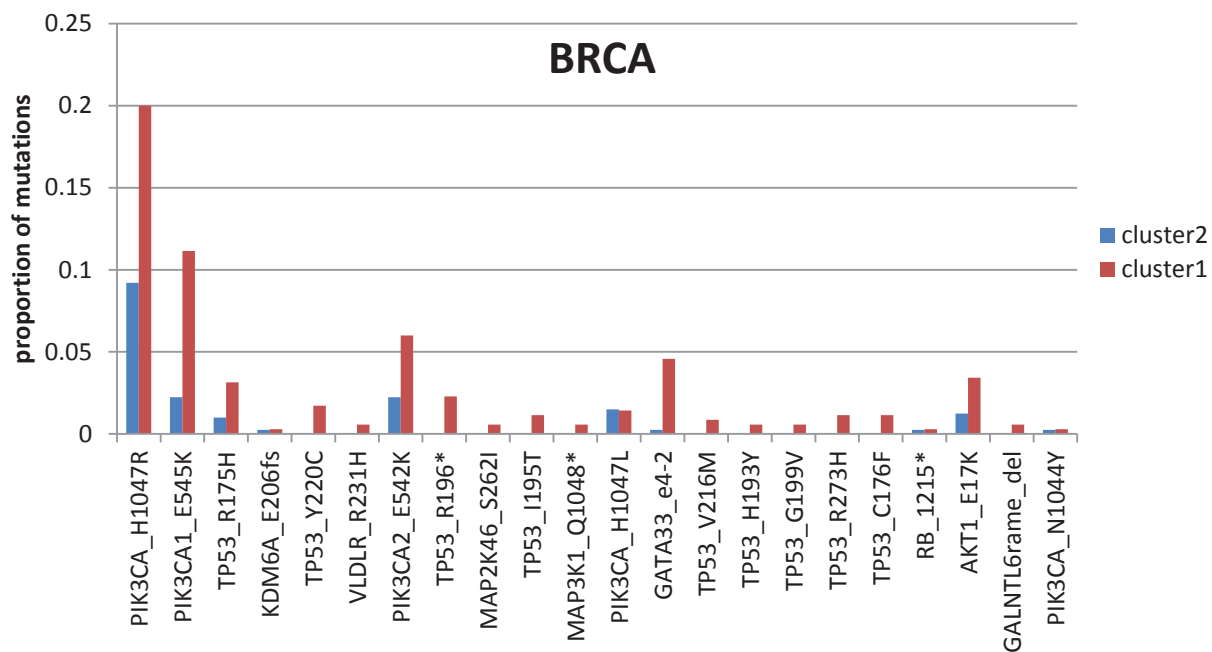

S13 Fig. The prevalence of mutations for the top 20 significant drivers across 2 clusters in BRCA.

Supplement: S13 Fig — (PDF) [file pone.0196939.s018.pdf]
