# Supplementary material for: Cancer driver mutation prediction through Bayesian integration of multi-omic data
Source: PLoS One. 2018 May 8;13(5):e0196939. doi: 10.1371/journal.pone.0196939 (PMC5940219; doi:10.1371/journal.pone.0196939)
Supplement: S15 Fig — (PDF) [file pone.0196939.s020.pdf]

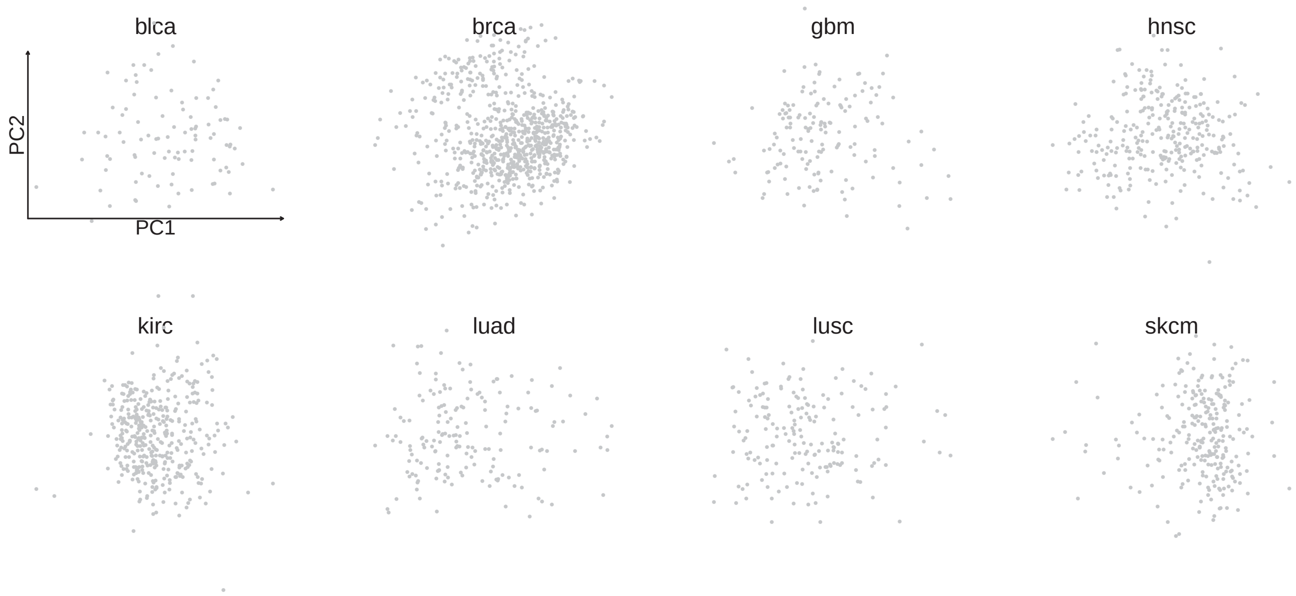

S15 Fig. Scatter plot of the first two principal components of gene expression of the 3,030 genes across 8 cancer types.
