# Supplementary material for: Cancer driver mutation prediction through Bayesian integration of multi-omic data
Source: PLoS One. 2018 May 8;13(5):e0196939. doi: 10.1371/journal.pone.0196939 (PMC5940219; doi:10.1371/journal.pone.0196939)
Supplement: S1 Table — (DOCX) [file pone.0196939.s021.docx]

Supplementary Table 1: Data summary of pan cancer 8 including mutation, expression and their patient numbers.

| **Data** | **recurrent Mutations(>=2)** | **Patients** | **Number of drivers predicted by rDriver** |
| --- | --- | --- | --- |
| BLCA | 227 | 95 | 104 |
| BRCA | 544 | 752 | 228 |
| GBM | 115 | 150 | 67 |
| HNSC | 493 | 299 | 182 |
| KIRC | 754 | 391 | 206 |
| LUAD | 416 | 169 | 180 |
| LUSC | 224 | 177 | 121 |
| SKCM | 7165 | 251 | 301 |
| total | 9938 | 2284 | 1389 |
