# Supplementary material for: Cancer driver mutation prediction through Bayesian integration of multi-omic data
Source: PLoS One. 2018 May 8;13(5):e0196939. doi: 10.1371/journal.pone.0196939 (PMC5940219; doi:10.1371/journal.pone.0196939)
Supplement: S2 Table — (DOCX) [file pone.0196939.s022.docx]

Supplementary Table 2: The genes carrying novel mutations of rDriver prediction in 8 cancer types, and their function annotation from literatures.

| gene | Functional annotation | disease | Reference |
| --- | --- | --- | --- |
| ARHGAP5 | promote cell spreading and migration by regulating RhoA | HCC/esophageal squamous cell | Gen et al., 2008 |
| C6 | related to HCC recurrence | HCC | Wang et al., 2016 |
| CDH6 | loosen the rigid organization, invasiveness, and act through TGB-beta downstream | Thyroid tumor | Sancisi, et al., 2013 |
| CNKSR1 | rare germline mutation through mutation burden | Breast and cervical cancer | Lu et al., 2016 |
| DHX9 | increasing H-DNA induced mutagenesis,related with genomic stability | Lymphomas | Jain et al., 2013 |
| PBX2 | interact with HOX protein, and increase gene transcription | Leukemia | Li et al., 2013 |
| PPP1R3A | muscule glycogen metabolism, eQTL and family | Muscle glycogen turnover | Savage et al., 2008 |
| RHEB | downstream and TSC1 and upstream of mTOR | Cancer | Inoki et al., 2003 |
| SERPINB13 | protease inhibitors, progression of HNSCC | HNSC | de Koning et al., 2009 |
| SON | proliferation and tumorigenicity of pancreatic cancer cell | Pancreatic | Furukawa et al., 2012 |
| ST14 | matriptase, eQTL | Brease cancer | Kauppinen et al., 2010 |
| STK39 | EQTL, non-small cell Lung cancer | LUSC | Huang et al., 2009 |
| TSHZ2 | tumor suppressor gene | Breast prostate cancer | Yamaoto et al., 2011 |
| UPF3a | nonsense mediated RNA decay | Cancer | Shum et al., 2016 |
| VLDLR | lipoprotein receptor, energy source of grouth | Gastric cancer | Takada et al., 2006 |
