# Supplementary material for: Cancer driver mutation prediction through Bayesian integration of multi-omic data
Source: PLoS One. 2018 May 8;13(5):e0196939. doi: 10.1371/journal.pone.0196939 (PMC5940219; doi:10.1371/journal.pone.0196939)
Supplement: S3 Table — (DOCX) [file pone.0196939.s023.docx]

Supplementary Table 3: The number of clonal or subclonal mutations in driver and passenger group across 8 cancer types.

| **Cancer** | **Type** | **Clonal** | **Subclonal** |
| --- | --- | --- | --- |
| BLCA | driver | 115 | 23 |
| BLCA | passenger | 53 | 28 |
| HNSC | driver | 229 | 55 |
| HNSC | passenger | 100 | 39 |
| SKCM | driver | 2381 | 277 |
| SKCM | passenger | 7191 | 1133 |
| GBM | driver | 89 | 22 |
| GBM | passenger | 14 | 13 |
| BRCA | driver | 429 | 54 |
| BRCA | passenger | 58 | 19 |
| LUSC | driver | 115 | 19 |
| LUSC | passenger | 82 | 26 |
| KIRC | driver | 57 | 20 |
| KIRC | passenger | 19 | 23 |
| LUAD | driver | 131 | 38 |
| LUAD | passenger | 48 | 30 |
