# Supplementary material for: Cancer driver mutation prediction through Bayesian integration of multi-omic data
Source: PLoS One. 2018 May 8;13(5):e0196939. doi: 10.1371/journal.pone.0196939 (PMC5940219; doi:10.1371/journal.pone.0196939)
Supplement: S4 Table — (DOCX) [file pone.0196939.s024.docx]

Supplementary Table 4: *In vitro* experimental functional validation of mutations using BA/F3 and MCF10A cell lines.

| **Gene** | **Mutations** | **Final_Call** | **DriverOrPassenger** | **rDriverPrediction** | **cancerTypeIncluded&Frequency** | **IPCT ( Therapeutical implication)** | **PolyPhen** | **SIFT** |
| --- | --- | --- | --- | --- | --- | --- | --- | --- |
| PIK3CA | N1044K | Strong activating | Driver | Passenger | KIRC,2 | Potential | possibly_damaging | deleterious |
| PIK3CA | C420R | Strong activating | Driver | Driver | BRCA,3, | Potential | probably_damaging | deleterious |
| PIK3CA | E453K | Moderate activating | Driver | Driver | BRCA,4 | NA | possibly_damaging | tolerated |
| PIK3CA | E542K | Moderate activating | Driver | Driver | HNSC,14,BLCA,2,BRCA,2,LUSC,3 | Potential | probably_damaging | deleterious |
| PIK3CA | E545A | No activating | Passenger | Driver | BRCA,2 | NA | probably_damaging | deleterious |
| PIK3CA | E545K | Strong activating | Driver | Driver | HNSC,20,GBM,2,BLCA,7,BRCA,48, | Potential | possibly_damaging | deleterious |
|  |  |  |  |  | LUSC,10, KIRC,3,LUAD,4 |  |  |  |
| PIK3CA | E726K | Weak activating | Driver | Driver | BRCA,8,LUSC,2 | NA | benign | tolerated |
| PIK3CA | H1047L | Strong activating | Driver | Driver | BRCA,11 | Potential | NA | NA |
| PIK3CA | H1047R | Strong activating | Driver | Driver | HNSC,7,BRCA,107,LUSC,2 | Potential | benign | deleterious |
| PIK3CA | E110del | Strong activating | Driver | Driver | BRCA,3 | NA | NA | NA |
| PIK3CA | K111del | Strong activating | Driver | Driver | BRCA,2 | NA | NA | NA |
| PIK3CA | M1004I | No activating | Passenger | Driver | BRCA,2 | NA | benign | deleterious |
| PIK3CA | M1043I | Moderate activating | Driver | Driver | BRCA,2 | NA | benign | deleterious |
| PIK3CA | M1043V | Strong activating | Driver | Driver | HNSC,2 | NA | benign | deleterious |
| PIK3CA | N345K | Strong activating | Driver | Driver | BRCA,11, | Potential | probably_damaging | tolerated |
| PIK3CA | Q546K | Strong activating | Driver | Driver | BRCA,4 | NA | possibly_damaging | tolerated |
| PIK3CA | Q546R | Strong activating | Driver | Driver | BRCA,5 | NA | probably_damaging | tolerated |
| PIK3CA | R88Q | Moderate activating | Driver | Driver | BRCA,2, | Potential | probably_damaging | deleterious |
| PIK3CA | G118D | Moderate activating | Driver | Driver | BRCA,2 | Potential | possibly_damaging | deleterious |
