# Supplementary material for: Cancer driver mutation prediction through Bayesian integration of multi-omic data
Source: PLoS One. 2018 May 8;13(5):e0196939. doi: 10.1371/journal.pone.0196939 (PMC5940219; doi:10.1371/journal.pone.0196939)
Supplement: S5 Table — (DOCX) [file pone.0196939.s025.docx]

Supplementary Table 5: The significance of survival separation of subgroups based on IPS and mRNA expression, respectively.

| Number of Cluster | 2 | 3 | 4 | 5 | 6 |
| --- | --- | --- | --- | --- | --- |
| BLCA |  |  |  |  |  |
| IPS | 0.6868068 | 0.3046231 | 0.751232 | 0.162465 | 0.37662 |
| 500 genes with most variable expression | 0.707397 | 0.721583 | 0.585121 | 0.926216 | 0.589085 |
| BRCA |  |  |  |  |  |
| IPS | 0.04098419 | 0.3514065 | 0.200351 | 0.231103 | 0.567857 |
| 500 genes with most variable expression | 0.6886043 | 0.09631112 | 0.38324 | 0.223867 | 0.06176 |
| GBM |  |  |  |  |  |
| IPS | 0.00436781 | 0.0028861 | 0.023468 | 0.126741 | 0.151784 |
| 500 genes with most variable expression | 0.1830921 | 0.6020441 | 0.351756 | 0.564078 | 0.87042 |
| HNSC |  |  |  |  |  |
| IPS | 0.02059438 | 0.06962168 | 0.003615 | 0.005484 | 0.041108 |
| 500 genes with most variable expression | 0.01542287 | 0.01702076 | 3.48E-05 | 0.001001 | 0.002748 |
| KIRC |  |  |  |  |  |
| IPS | 5.71E-01 | 2.33E-01 | 3.70E-01 | 5.75E-01 | 3.90E-01 |
| 500 genes with most variable expression | 1.12E-11 | 1.19E-06 | 3.05E-12 | 2.91E-11 | 1.98E-10 |
| LUAD |  |  |  |  |  |
| IPS | 0.8765209 | 0.2371253 | 0.538356 | 0.148018 | 0.301975 |
| 500 genes with most variable expression | 0.5038425 | 0.1168733 | 0.004343 | 0.022043 | 0.00524 |
| LUSC |  |  |  |  |  |
| integrative Score | 0.3128678 | 0.3870523 | 0.355074 | 0.447466 | 0.668531 |
| 500 genes with most variable expression | 0.5120698 | 0.2869964 | 0.15739 | 0.21234 | 0.450457 |
| SKCM |  |  |  |  |  |
| IPS | 0.06670607 | 4.07E-02 | 2.15E-05 | 8.36E-05 | 1.00E-03 |
| 500 genes with most variable expression | 0.02721995 | 7.10E-08 | 1.35E-05 | 4.87E-05 | 1.55E-06 |
