# Supplementary material for: Cancer driver mutation prediction through Bayesian integration of multi-omic data
Source: PLoS One. 2018 May 8;13(5):e0196939. doi: 10.1371/journal.pone.0196939 (PMC5940219; doi:10.1371/journal.pone.0196939)
Supplement: S11 Fig — Stars indicate significance of enrichment of the related frequency occurring in CGC in driver group. (**<0.01, *p<0.05; Fisher’s exact test). (PDF) [file pone.0196939.s016.pdf]

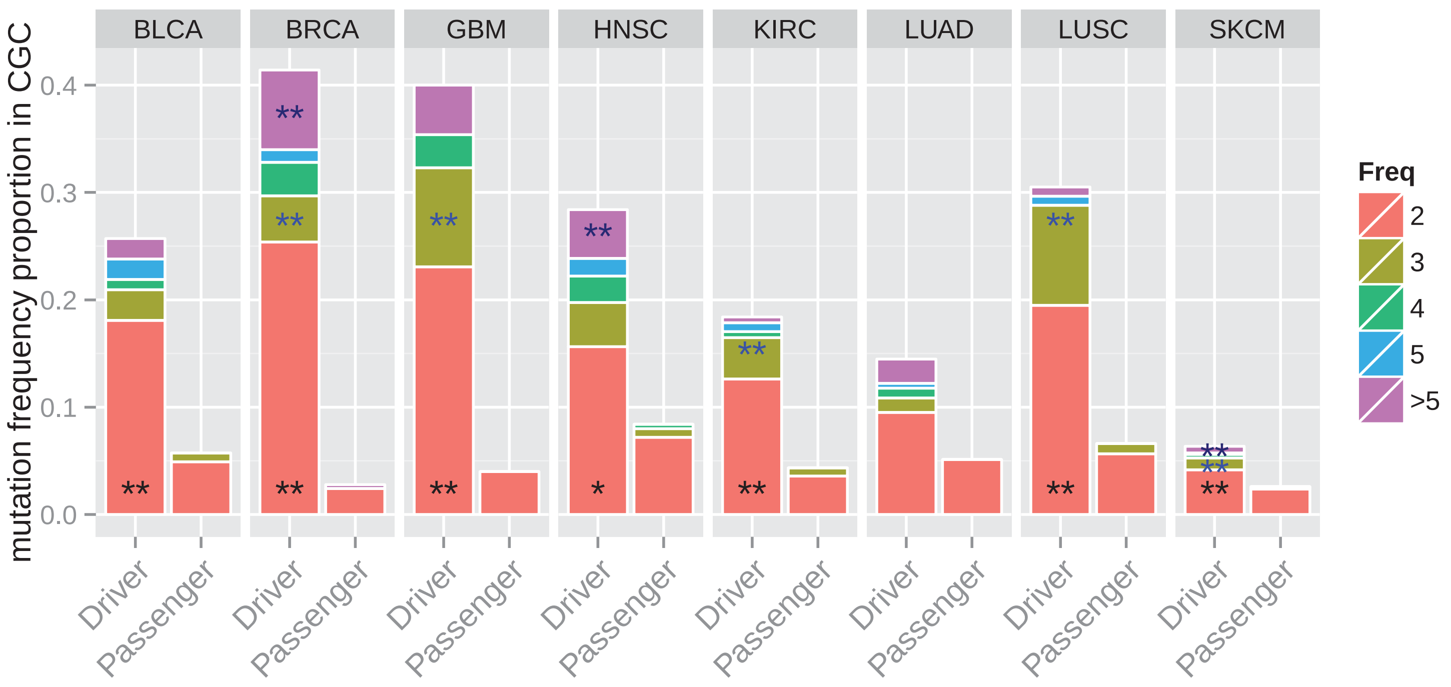

S11 Fig. The proportion of mutations that occur to cgc is different between the two groups. Stars indicate significance of enrichment of the related frequency occurring in cgc in driver group. (**<0.01, *p<0.05; Fisher's exact test).
